# Supplementary figures and images for: Human herpesvirus 8 molecular mimicry of ephrin ligands facilitates cell entry and triggers EphA2 signaling
Source: PLoS Biol. 2021 Sep 9;19(9):e3001392. doi: 10.1371/journal.pbio.3001392 (PMC8454987; doi:10.1371/journal.pbio.3001392)

S1 Fig: Schematic representation of HHV-8 entry into cells

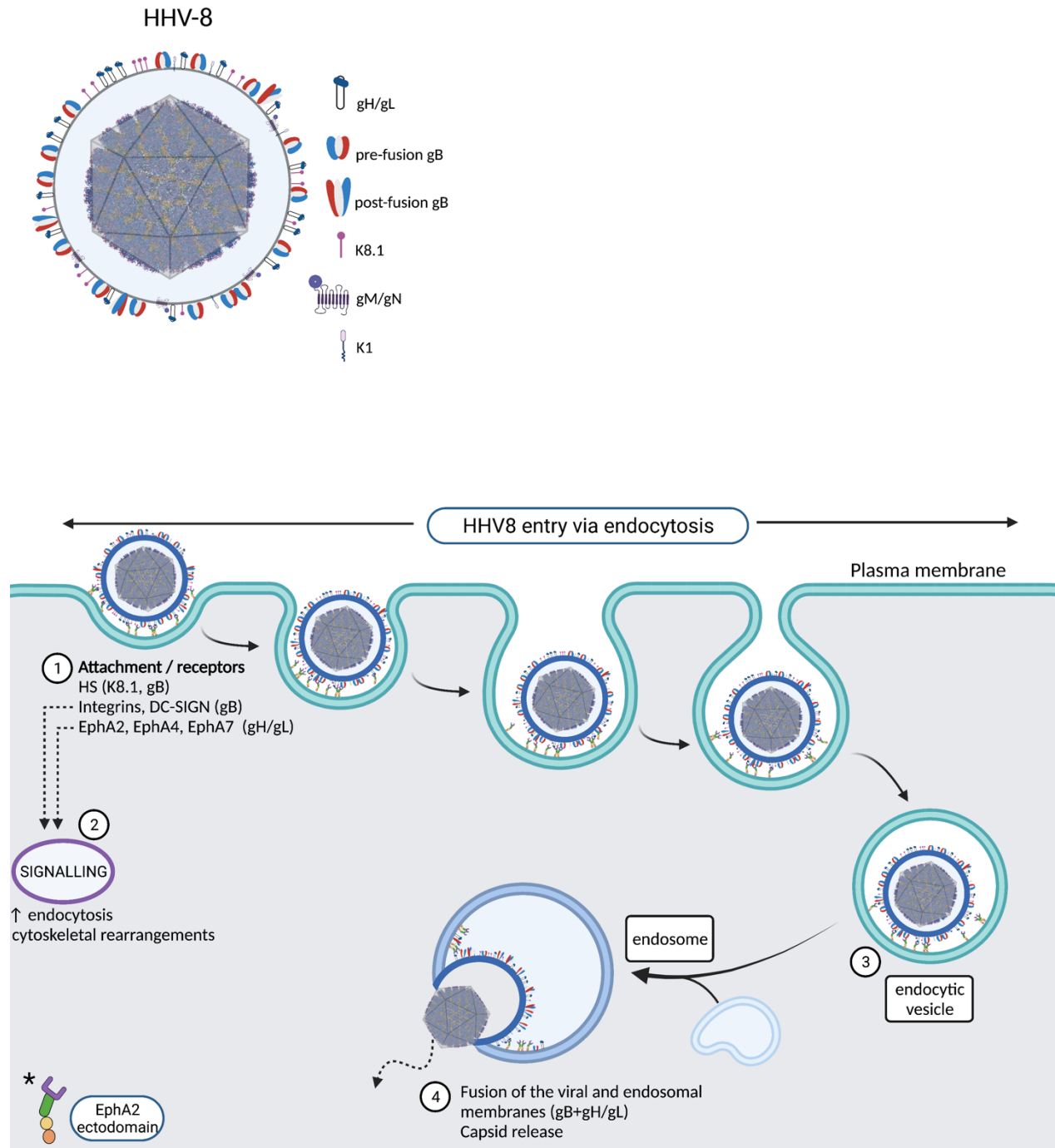

Supplement: S1 Fig — Major envelope glycoproteins are indicated on the surface of the virus particle. The initial attachment of HHV-8 to the cells is mediated by glycoproteins K8.1 and gB, which bind to heparan sulfate via multiple low-affinity interactions. Specific interactions—with integrins and receptors from EphA family of tyrosine kinases—determine cell tropism and are mediated by gB and gH/gL, respectively. Viral glycoproteins (gB, gH, K8.1, K.1) and EphA2 on the host cell, which are all single-pass transmembrane proteins, are depicted only as ectodomains for clarity reasons. The figure was created in BioRender.com. gB, glycoprotein B; gH/gL, glycoproteins H and L; HHV-8, human herpesvirus 8. (PDF) [file pbio.3001392.s001.pdf]

S2 Fig: Oligomeric assemblies formed by EphA2 ectodomains

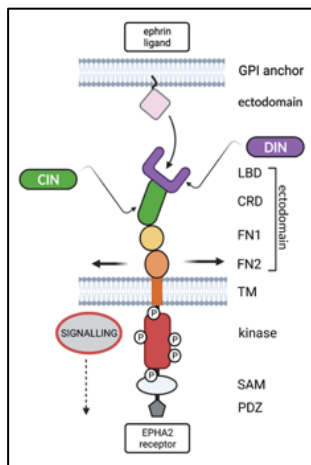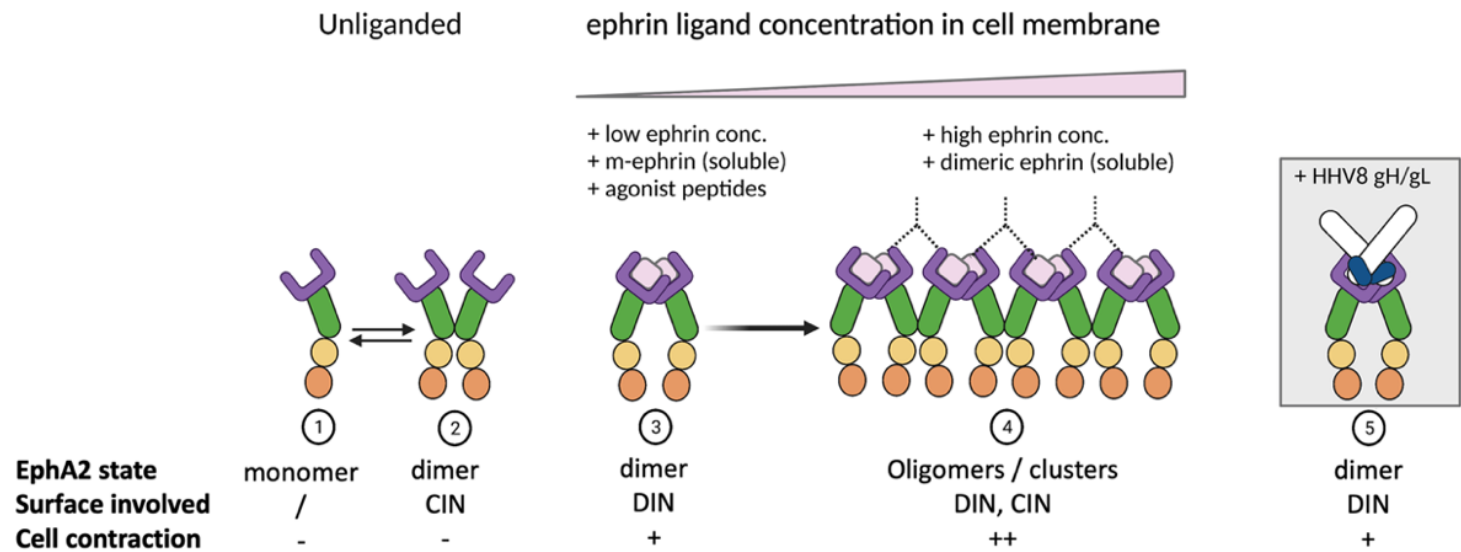

Supplement: S2 Fig — (Inlet) The full-length EphA2 and ephrin ligand are shown as anchored in 2 opposing membranes. The EphA2 ectodomain is made of, going from the N to C terminus: the LBD colored in purple, the CRD in green, and 2 FN-like domains in yellow and orange, respectively. The EphA2 DIN in the LBD and the CIN in the CRD are indicated. In the absence of ligand, EphA2 exists in an equilibrium between monomers (1) and dimers (2), with the unliganded EphA2 dimers stabilized via the CIN [1]. (3) At low ligand concentration or in the presence of soluble, m-ephrin ligands or agonist peptides [2], each Eph receptor interacts with 2 ephrin molecules—its cognate ligand with high affinity and via low affinity interactions with ephrin from the other complex, forming the so-called “tetrameric assembly” made of 2 receptor (EphA2 dimer stabilized via DIN) and 2 ligand molecules. (4) At higher ligand concentrations, emulated by addition of dimeric or preclustered soluble ephrin ligands, the EphA2 molecules from the tetrameric assembly interact with EphA2 from other tetramers via the CIN, giving rise to larger oligomeric structures, i.e., clusters. (5) HHV-8 gH/gL is drawn as gray/blue rectangles. Data presented in this manuscript demonstrate that soluble gH/gL induces formation of EphA2 dimers stabilized via DIN, similar to the effect of m-ephrinA2 or agonist peptides (3). The figure was created in BioRender.com. CIN, clustering surface; CRD, cysteine-rich domain; DIN, dimerization interface; Eph, erythropoietin-producing human hepatocellular carcinoma cell line; ephrin, Eph family receptor interacting protein; FN, fibronectin; gH/gL, glycoproteins H and L; HHV-8, human herpesvirus 8; LBD, ligand-binding domain. (PDF) [file pbio.3001392.s002.pdf]

S3 Fig: Secondary structure topology diagram of EphA2 LBD, gL and ephrin-A1

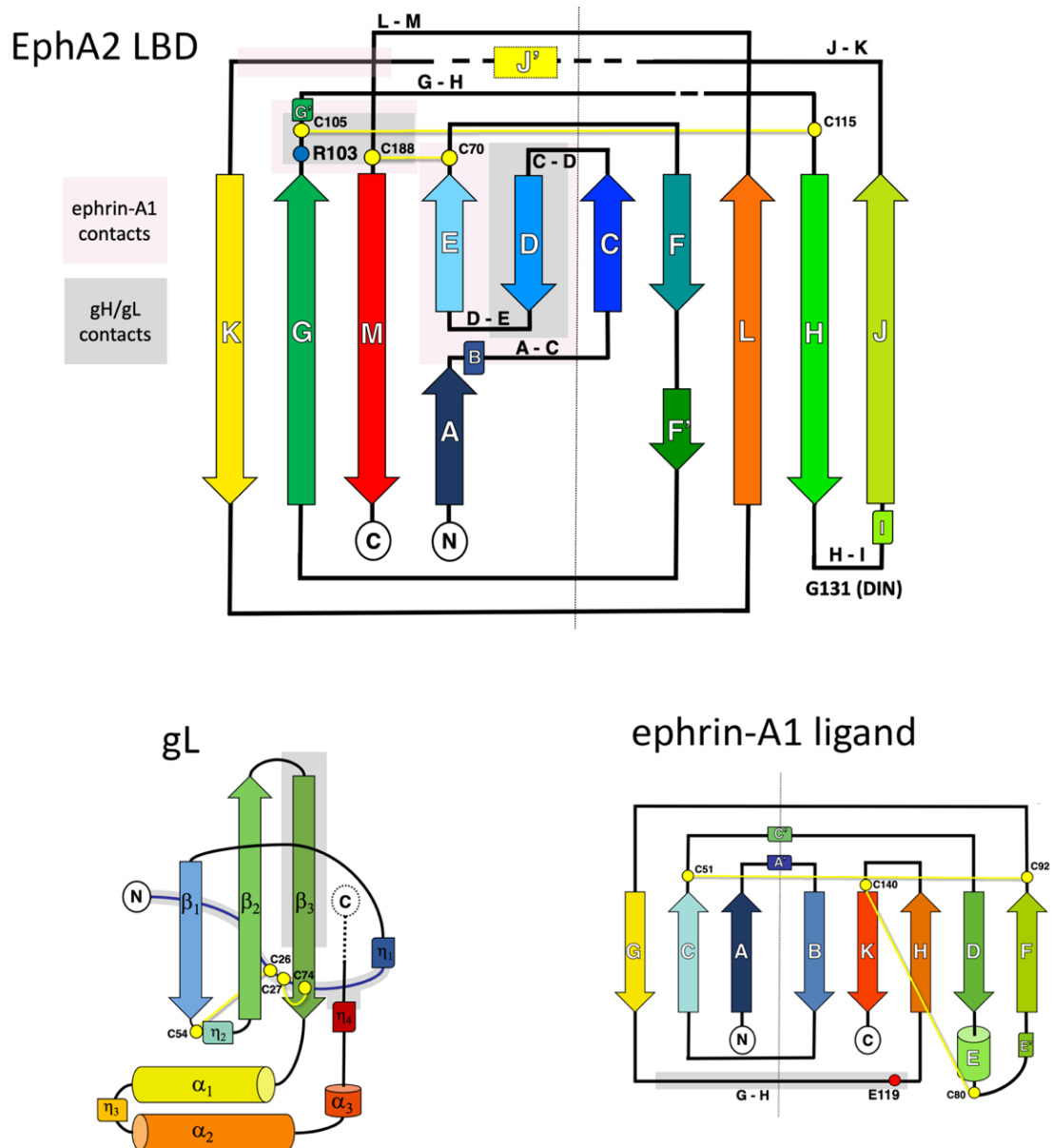

Supplement: S3 Fig — Secondary structure elements are represented by arrows (β-strands), rectangles (α-helices), and rounded rectangles (η helices (B, I, J’)). The dashed lines indicate regions not resolved in the structures. The vertical dotted lines designate the two 5-stranded β-sheets adopting a jelly roll fold in EphA2 LBD and a 3- and 5-stranded sheets forming a β-sandwich in ephrin-A1. The conserved residues R103EphA2 and E119ephrin-A1, which are important for high-affinity interaction, are represented as red and blue circles, respectively. Cysteine residues establishing disulfide bonds (yellow lines) are represented with yellow circles. The secondary structure diagrams for EphA2 LBD and HHV-8 gL are drawn based on the structure presented in this paper (PDB 7B7N), while ephrin-A1 ligand was represented as in the structure (PDB 3HEI) [3]. EphA2 LBD—gray and pink shaded areas indicate the structural elements involved in interactions with gH/gL and ephrin-A1, respectively. The ephrin uses an 18-residue long and mostly hydrophobic loop that connects strands G and H—the GHephrin loop—for insertion into a complementary hydrophobic cavity presented at the surface of the receptor EphA2 LBD [4]. The GHephrin loop carries a conserved E119ephrin-A1 (red circle) that establishes polar interactions, critical for high-affinity binding, with a strictly conserved R103EphA2 (blue circle) on the loop connecting strands G and H in EphA receptors, designated also as a GH loop (GHEphA2) [3]. In gL and ephrin-A1, gray shaded areas highlight the structural elements involved in interactions with EphA2 LBD. DIN, dimerization interface; ephrin, Eph family receptor interacting protein; gH/gL, glycoproteins H and L; HHV-8, human herpesvirus 8; LBD, ligand-binding domain. (PDF) [file pbio.3001392.s003.pdf]

S4 Fig: EphA2 LBD binds to HHV-8 gH/gL in 1:1 stoichiometry

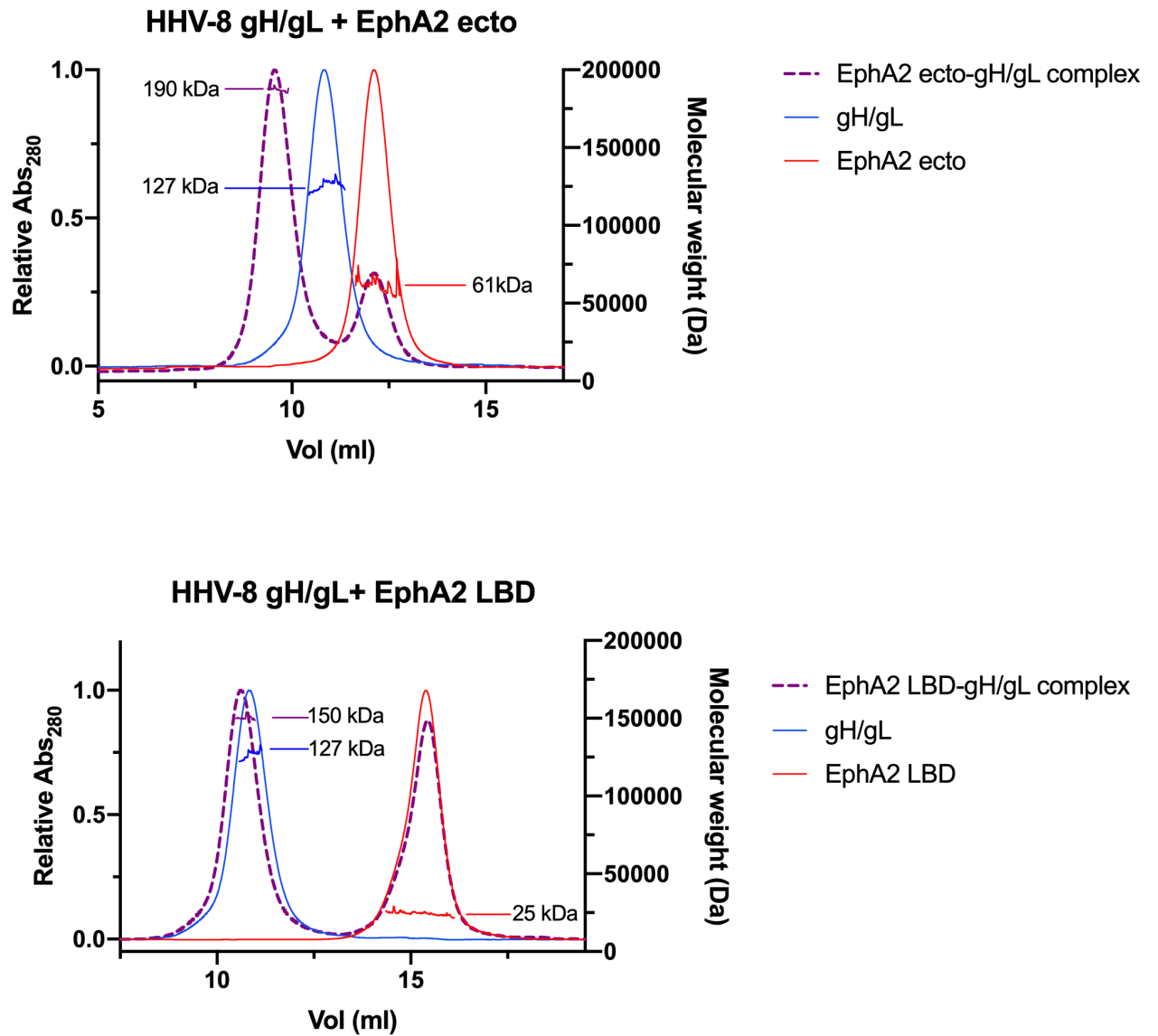

Supplement: S4 Fig — SEC-MALS traces are shown for HHV-8 gH/gL alone (blue curve), EphA2 ectodomain or LBD alone (red curves), and gH/gL mixed with EphA2 (purple, dashed curve). Molecular weights are indicated on the chromatograms, demonstrating that the tertiary complexes are composed of 1 molecule of HHV-8 gH/gL bound to 1 molecule of EphA2 ectodomain or EphA2 LBD. The underlying data can be found in S2 Data. gH/gL, glycoproteins H and L; HHV-8, human herpesvirus 8; LBD, ligand-binding domain; SEC-MALS, size exclusion chromatography coupled with multi-angle light scattering. (PDF) [file pbio.3001392.s004.pdf]

S6 Fig: Analyses of gH/gL and ephrin-A1 interfaces with EphA2 LBD

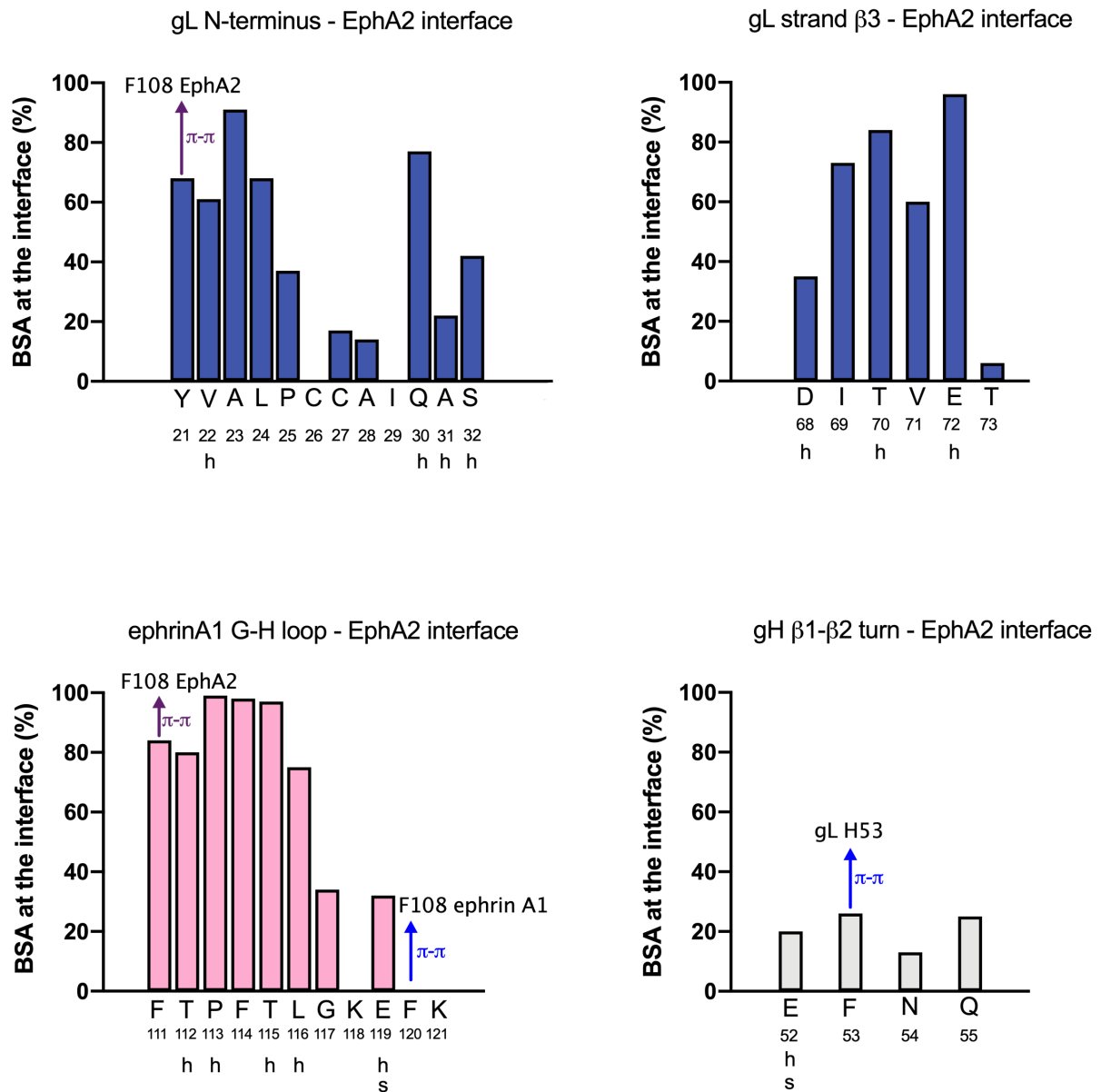

Supplement: S6 Fig — The BSA is presented as % of the total residue surface and is plotted for each residue indicated by a letter and number on the x-axis, for each given interface. The residues participating in hydrogen and salt bridge bonds are marked with “h” and “s,” respectively. The residues involved in pi–pi interactions are indicated with blue arrows. The underlying data can be found in S2 Data. BSA, buried surface area; ephrin, Eph family receptor interacting protein; gH/gL, glycoproteins H and L; LBD, ligand-binding domain. (PDF) [file pbio.3001392.s006.pdf]

S7 Fig: Glycosylation of HHV-8 gL variants

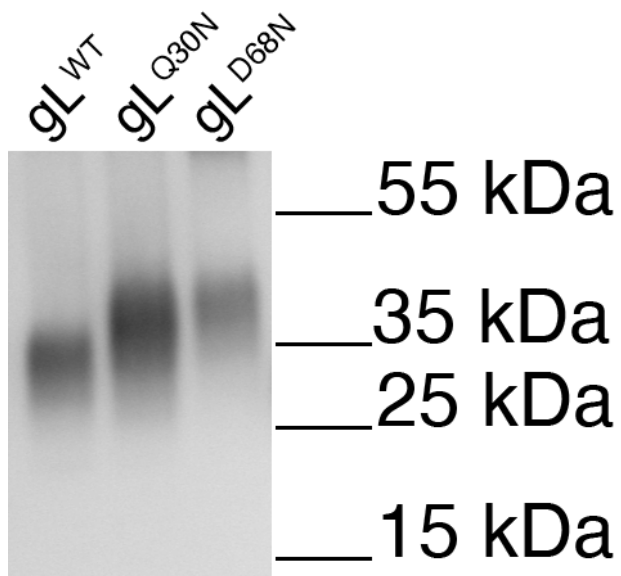

Supplement: S7 Fig — Aliquots of the purified gH/gLWT, gH/gLQ30N, and gH/gLD68N variants were analyzed by SDS-PAGE and western blotting to detect the DST affinity attached to the C terminus of gL. The higher molecular weight on the 2 variants is indicative of the presence of oligosaccharides at the newly introduced gL N-glycosylation sites, Q30N and D68N. DST, double-strep tag; HHV-8, human herpesvirus 8. (PDF) [file pbio.3001392.s007.pdf]

S8 Fig: Illustration of the BLI setup

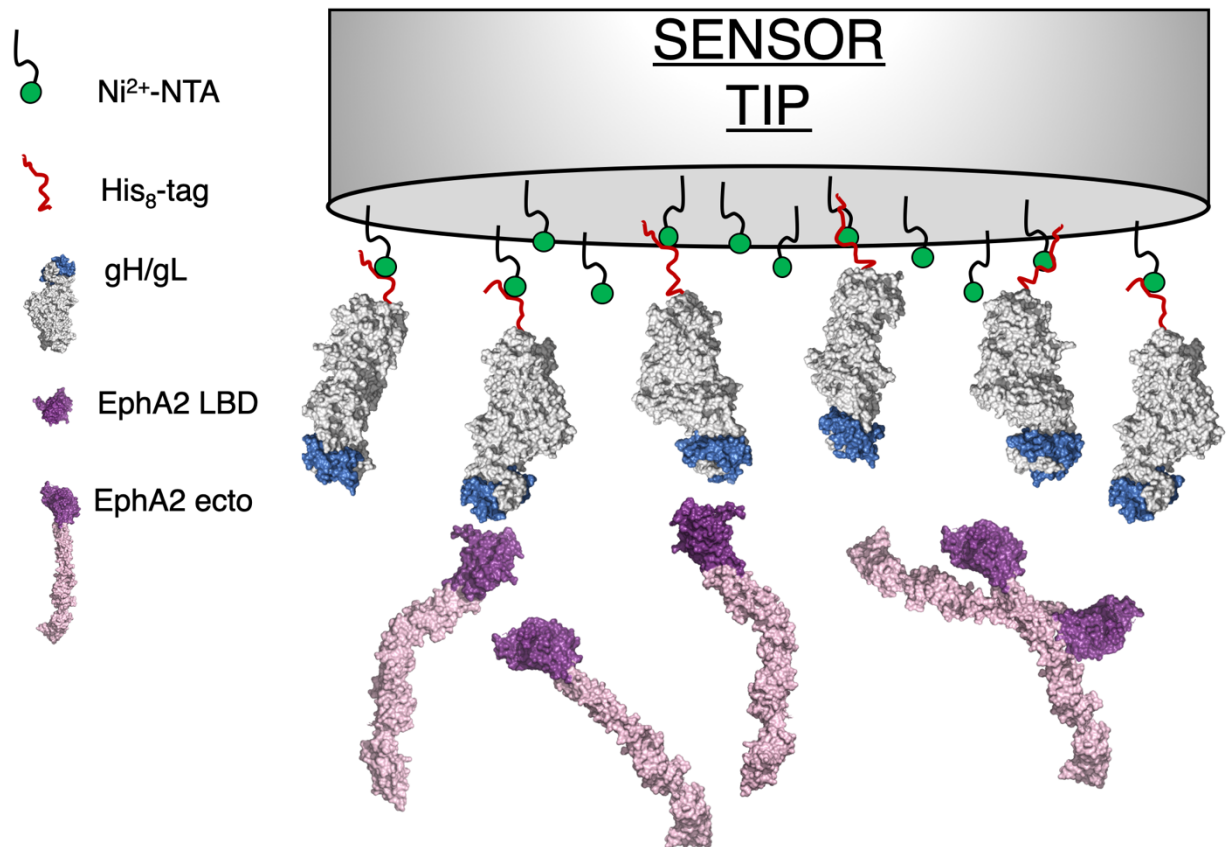

Supplement: S8 Fig — HHV-8 gH/gL is loaded onto the NTA-Ni2+ sensors via a histidine tag attached to the gH C terminus located at the opposite side from the gL and the EphA2 binding site. BLI, Biolayer interferometry; gH/gL, glycoproteins H and L; HHV-8, human herpesvirus 8; LBD, ligand-binding domain. (PDF) [file pbio.3001392.s008.pdf]

S9 Fig: Binding of WT gH/gL and EphA2 at low pH and in inverted system

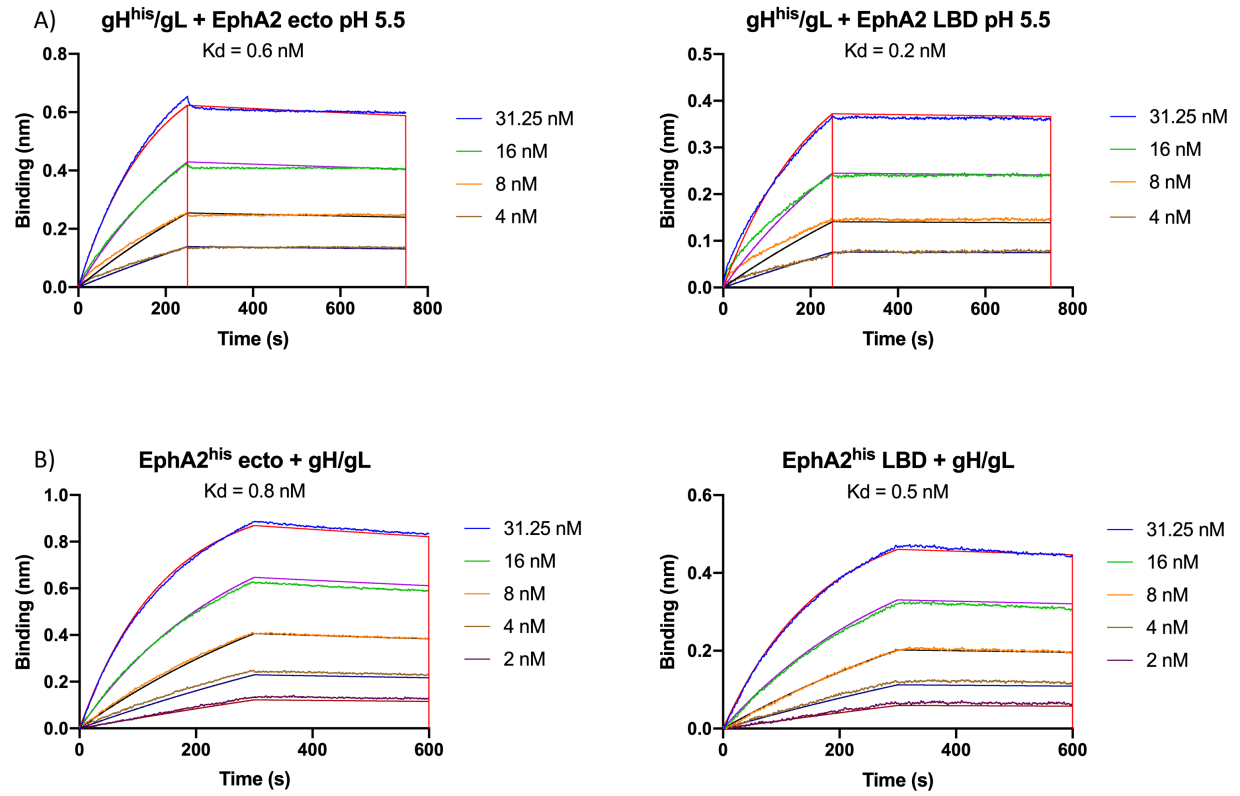

Supplement: S9 Fig — BLI sensorgrams obtained for (A) interactions between immobilized gH/gL and EphA2 ectodomain/LBD at pH 5.5 and (B) interactions between immobilized EphA2 ectodomain/LBD and gH/gL at pH 7.5. Immobilization was done via a histidine tag on gH/gL (panel A) or EphA2 (panel B). The underlying data can be found in S2 Data. BLI, Biolayer interferometry; gH/gL, glycoproteins H and L; LBD, ligand-binding domain; WT, wild-type. (PDF) [file pbio.3001392.s009.pdf]

# S11 Fig: Alignment of gL sequences from gammaherpesviruses

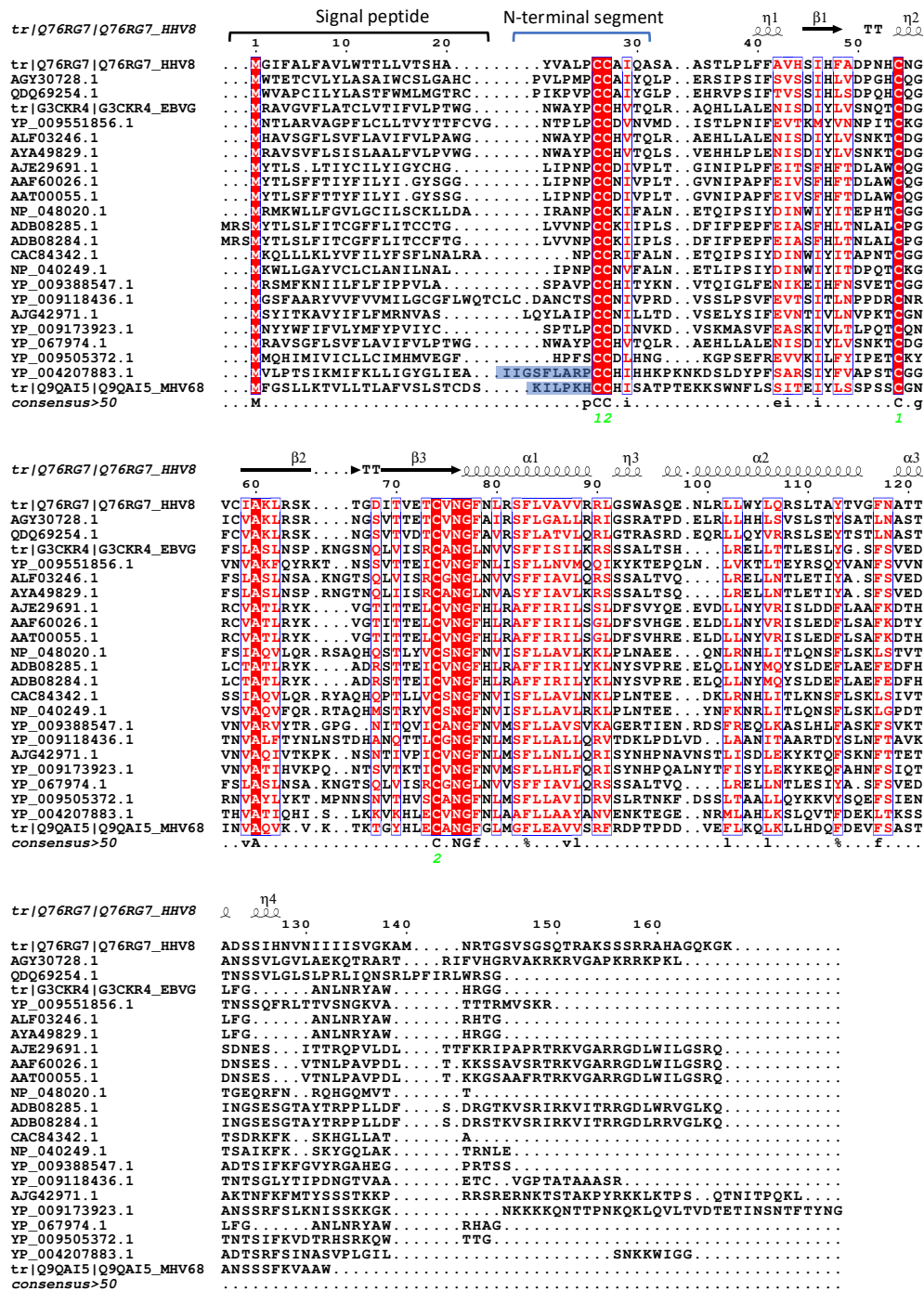

Supplement: S11 Fig — The HHV-8 gL sequence is placed on the top. The N termini in 2 rodent gLs (Cricetid gammaherpesvirus 2, accession number YP_004207883.1, and Murine gammaherpesvirus 68, accession number Q9QAI5_MHV68) contain positively charged residues and are shaded in blue on the bottom of the alignment. Secondary structure elements are indicated above the sequences, and the disulfide bridges (green letters) and consensus sequence below. The alignment was generated by Clustal Omega [7] and plotted by ESPript [8]. HHV-8, human herpesvirus 8. (PDF) [file pbio.3001392.s011.pdf]

S12 Fig: EphA2 assemblies and contacts observed in the crystal

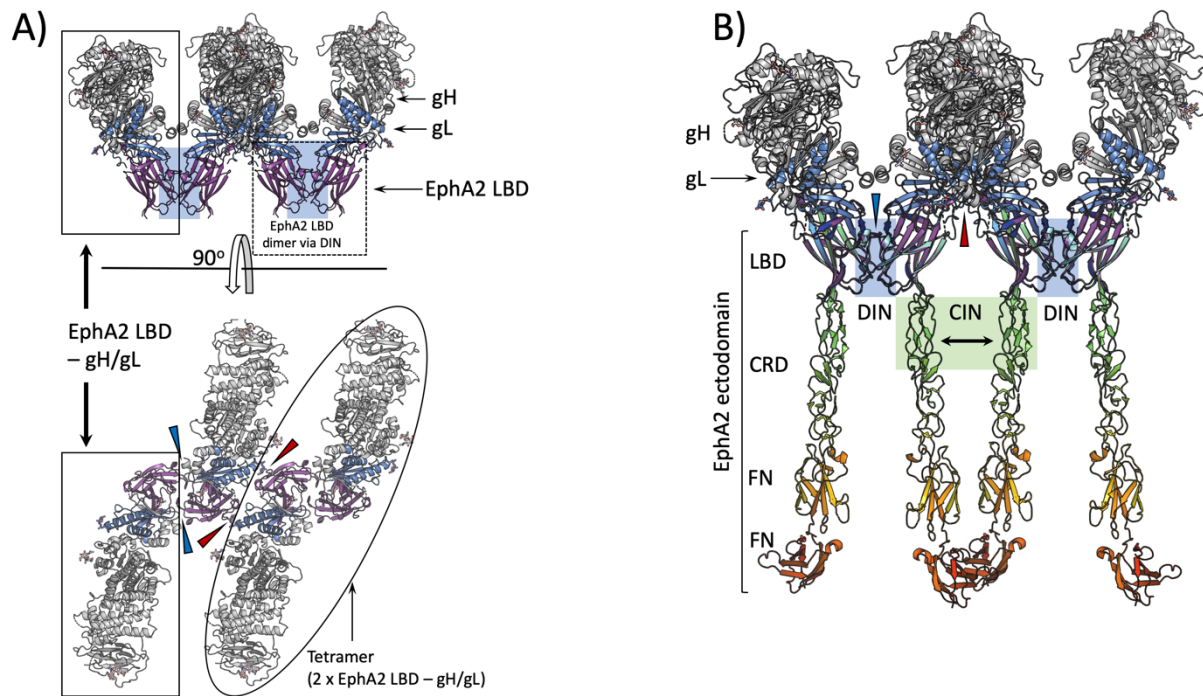

Supplement: S12 Fig — (A) Crystal packing of EphA2 LBD–gH/gL complexes. The solid line rectangles indicate a single tertiary complex (gH/gL-EphA2 LBD). The EphA2 LBD dimer formed via DIN (blue shaded box) is enclosed with a dashed line rectangle. Bottom panel shows the top view; a tetramer formed of 2 gH/gL and 2 EphA2 LBD molecules is indicated with an oval shape. The blue triangles indicate absence of contacts between gH/gL of one tertiary complex with the LBD from the adjacent complex molecule within the same tetramer. Red triangles point to the sites of contacts of gL with LBD from another tetrameric assembly. The latter contacts are formed between the NAG moiety at the N118 of gL and res C115 of EphA2 LBD (1 HB). (B) The structure of the EphA2 ectodomain (PDB 2X10, rainbow colors) was superimposed onto the EphA2 LBD bound to gH/gL to indicate the putative location of the EphA2 domains downstream from LBD in this kind of arrangement. The DIN and CIN are marked with the blue and green boxes, respectively. The distance between CIN, imposed by gH/gL packing, is too large for contacts to be established and drive aggregation of EphA2 dimers into larger oligomers via the CIN. CIN, clustering surface; CRD, cysteine-rich domain; DIN, dimerization interface; FN, fibronectin; gH/gL, glycoproteins H and L; LBD, ligand-binding domain. (PDF) [file pbio.3001392.s012.pdf]

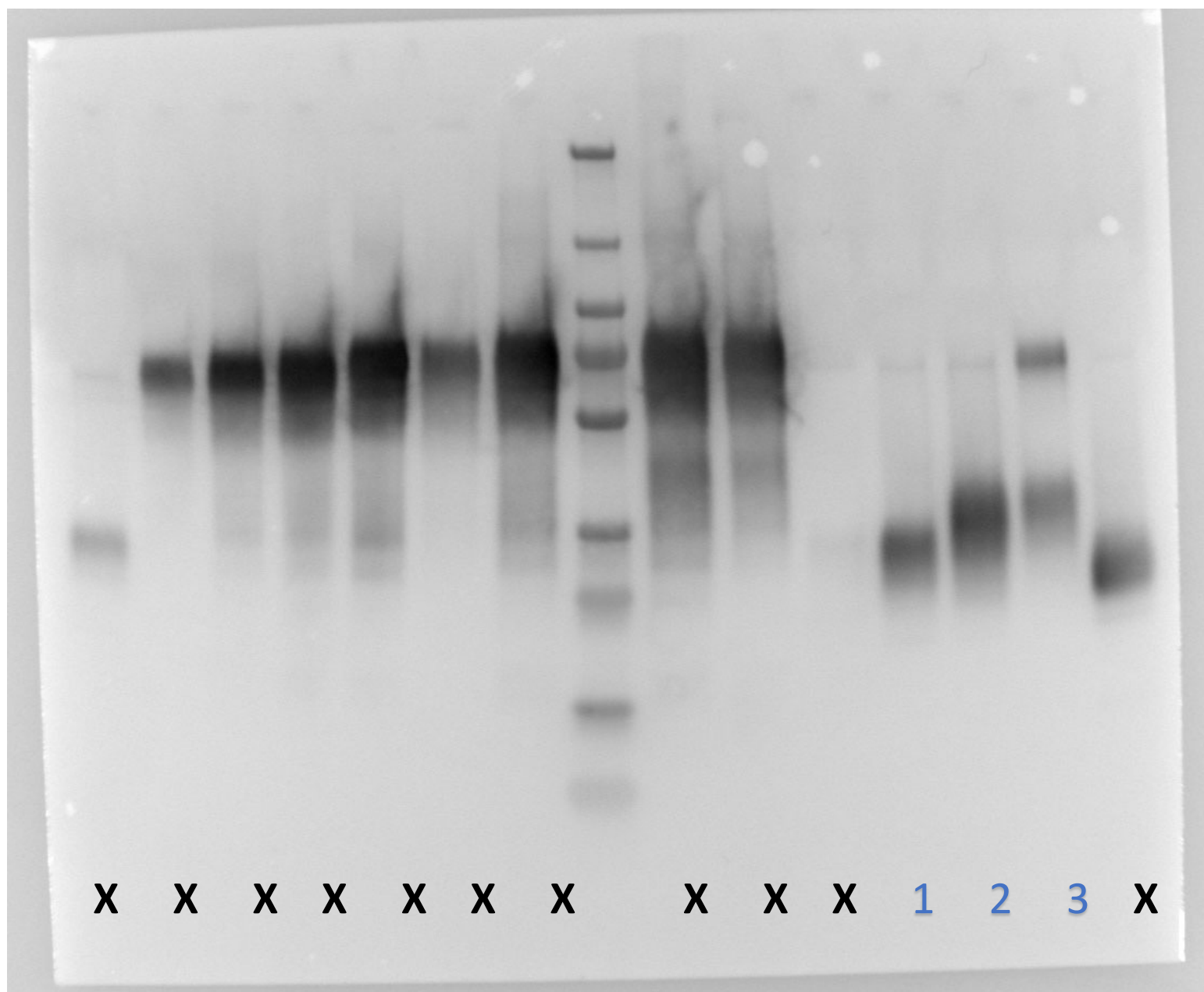

Supplement: S1 Raw Data — Lanes 1, 2, and 3 designated the gH/gL samples analyzed for the purposes of S7 Fig. The signs “X” designate samples irrelevant for the figure. (PDF) [file pbio.3001392.s020.pdf]
