# Supplementary material for: Human herpesvirus 8 molecular mimicry of ephrin ligands facilitates cell entry and triggers EphA2 signaling
Source: PLoS Biol. 2021 Sep 9;19(9):e3001392. doi: 10.1371/journal.pbio.3001392 (PMC8454987; doi:10.1371/journal.pbio.3001392)
Supplement: S5 Fig — (A) Available structures of the gH/gL complexes are shown, with the corresponding PBD access numbers indicated below. (B) The structures of HHV-8 gH/gL bound to EphA2 LBD, reported by Su and colleagues [5] and us, shown separately—left and central panel, respectively—and as a superimposition of the 2 structures to indicate the disposition of the gH molecule past the hinge helix. The structural alignments were performed using Dali Pairwise Structure Comparison server [6]. The gH domains were defined using the following HHV-8 gH assignment: domain I residues 35–87, domain II residues 88–365, domain III residues 366–553, and domain IV residues 554–696. Because of the variability in the length of the gH DI among different herpesviruses and poor or no conservation at the amino acid level, the hinge/linker helix was used as a demarcation point for the boundary between gH DI and DII. Z-scores are calculated as reported in (6) and indicate structural similarity. “RMSD” is the average distance deviation between the aligned Cα atoms in Å; “lali” refers to the number of aligned, i.e., structurally equivalent residues; and “nres” is the total number of residues in the target protein. The sequence identity (“id”) is computed from the structural alignment as the ratio between the number of structurally aligned residues and the total number of residues. CMV, cytomegalovirus; EBV, Epstein–Barr virus; gH/gL, glycoproteins H and L; HHV-8, human herpesvirus 8; HSV-2, herpes simplex virus 2; LBD, ligand-binding domain; RMSD, root-mean-square deviation; VZV, varicella-zoster virus. (PDF) [file pbio.3001392.s005.pdf]

S5 Figs: Structural comparison of gH/gL from gamma- (HHV-8, EBV), beta- (CMV) and alpha-herpesviruses (HSV-2 and VZV)

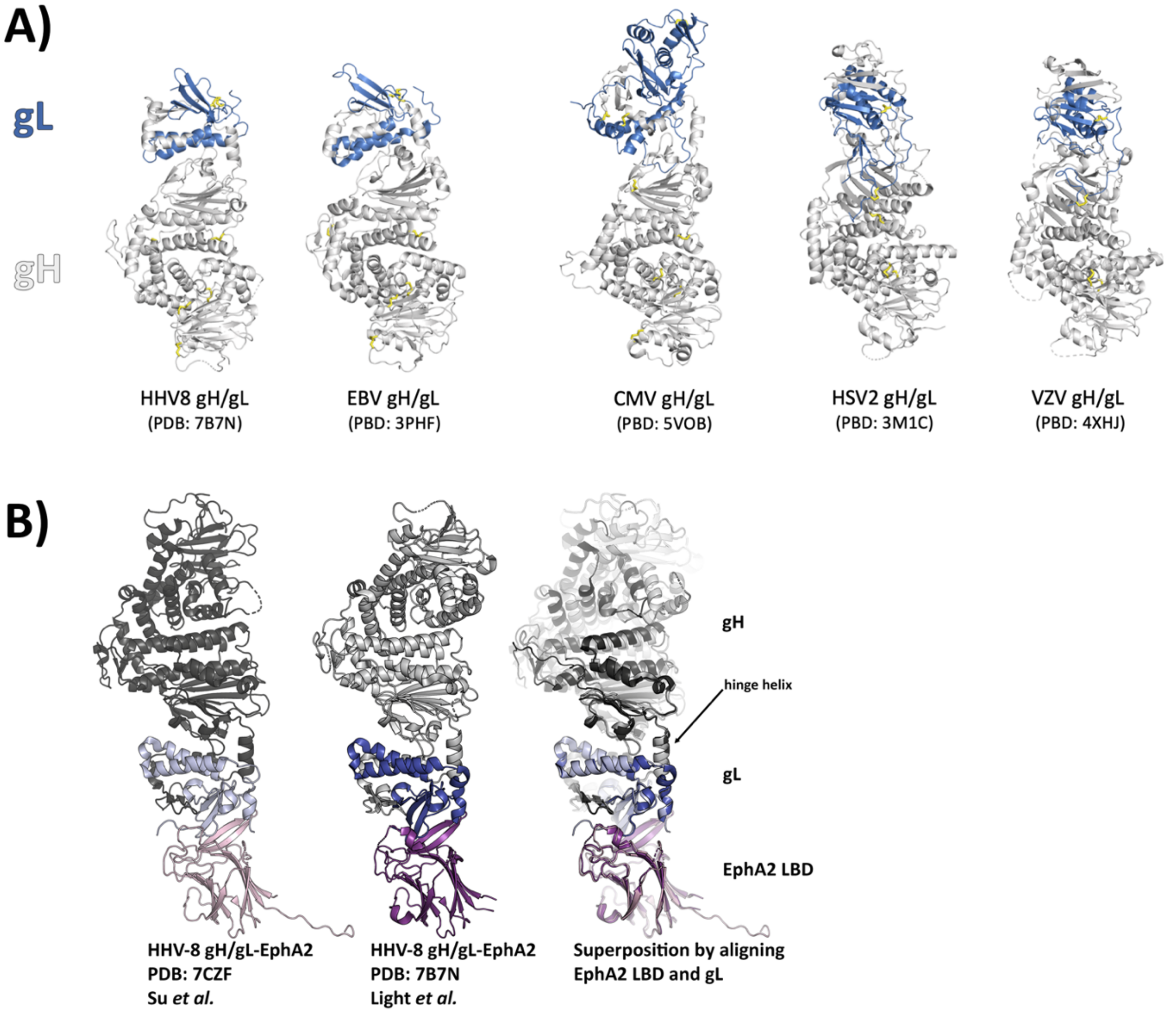

		EBV				CMV				HSV-2				VZV				HHV-8			
PDB:		3PHF				5VOB				3M1C				4XHJ				7CZF			
HHV-8		Z	rmsd	lali/ nres	%id	Z	rmsd	lali/ nres	%id	Z	rmsd	lali/ nres	%id	Z	rmsd	lali/ nres	%id	Z	rmsd	lali/ nres	%id
gH	DI	3.1	2.7	43/ 46	26	-	-	-	-	-	-	-	-	-	-	-	-	7.9	0.9	52/ 52	87
	DII	28.1	2.7	262/ 279	21	19.3	3.1	230/ 282	17	10.2	3.3	148/ 210	9	10.1	3.3	163/ 206	7	38.8	1.2	268/ 278	100
	DIII	18.4	2.3	171/ 185	25	14.6	2.8	154/ 168	14	13.6	2.8	152/ 179	18	12.0	3.1	150/ 184	18	22.7	1.4	158/ 168	95
	DIV	17.3	2.4	132/ 143	39	15.2	2.3	129/ 145	22	15.1	2.3	125/ 148	20	14.5	2.5	131/ 150	19	22.7	1.2	138/ 146	95
gL		12.3	1.7	97/ 108	24	2.6	3.8	75/ 237	15	6.6	3.2	94/ 146	11	6.3	3.3	93/ 132	13	19.1	0.6	107/ 109	99
