# Supplementary material for: Human herpesvirus 8 molecular mimicry of ephrin ligands facilitates cell entry and triggers EphA2 signaling
Source: PLoS Biol. 2021 Sep 9;19(9):e3001392. doi: 10.1371/journal.pbio.3001392 (PMC8454987; doi:10.1371/journal.pbio.3001392)

S10 Fig: FSI-FRET data: Proximity-corrected FRET efficiencies, donor concentrations, and acceptor concentrations.

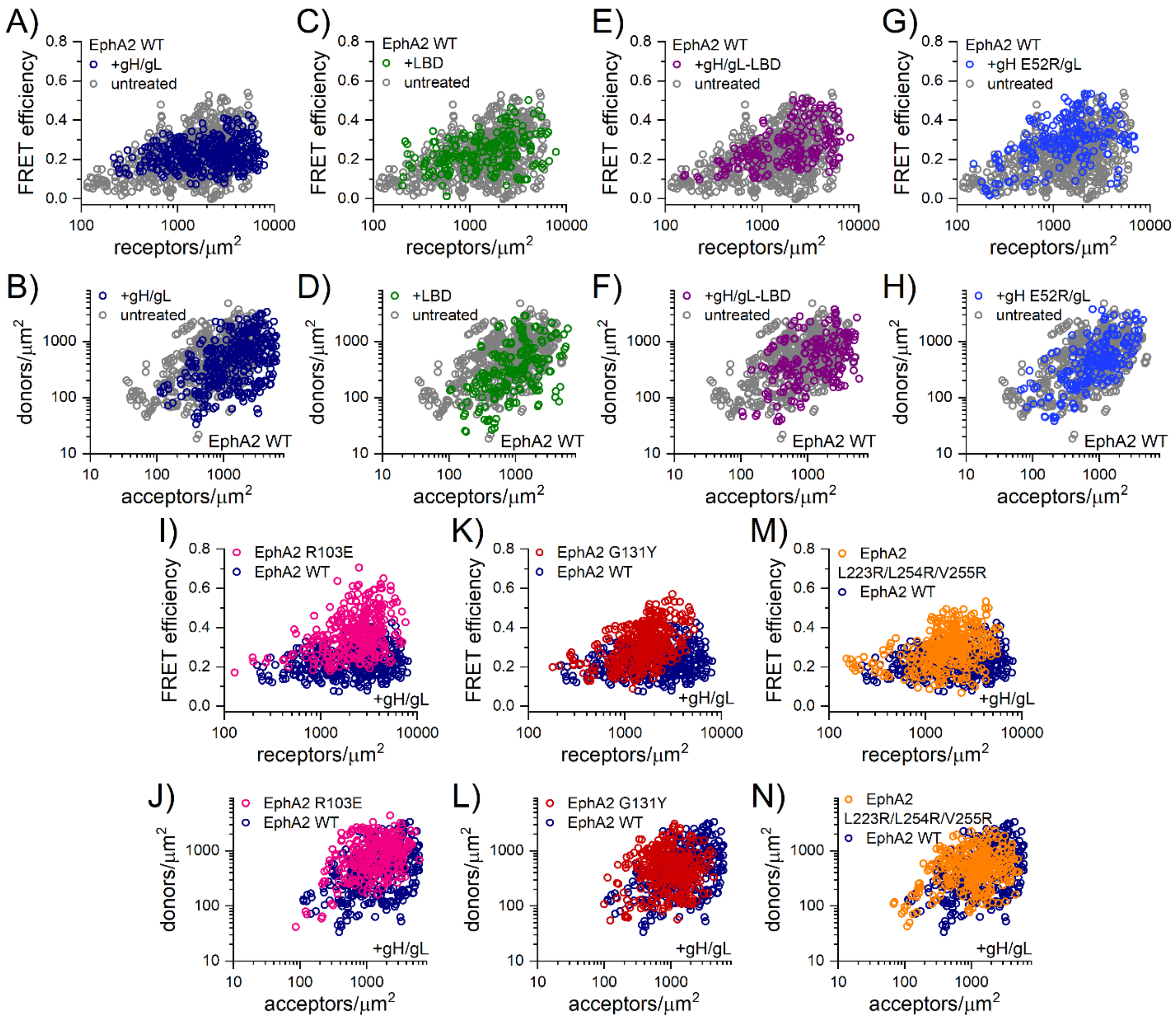

Supplement: S10 Fig — The FSI-FRET method determines the FRET efficiencies, the concentration of donor-tagged EphA2 (EphA2-mTURQ), and the concentration of acceptor-tagged EphA2 (EphA2-eYFP) at the plasma membrane of live HEK293T cells. Each FRET dataset is combined from at least 10 independent experiments. The FRET efficiencies were corrected for the nonspecific “proximity FRET” contribution and are plotted as a function of the measured receptor concentration (EphA2-mTURQ+EphA2-eYFP concentrations). The proximity-corrected FRET efficiencies and the donor and acceptor concentrations were measured for the following conditions: (A, B) EphA2 WT +gH/gL, (C, D) EphA2 WT +LBD, (E, F) EphA2 WT +gH/gL-LBD, (G, H) EphA2 WT +gH E52R/gL, (I, J) EphA2 R103E +gH/gL, (K, L) EphA2 G131Y +gH/gL, and (M, N) EphA2 L223R/L254R/V255R +gH/gL. The data in (A–H) are compared to EphA2 WT data in the absence of ligand (untreated), which was previously reported (2). The data in (I–N) are compared to EphA2 WT in the presence of gH/gL (from (A, B)). The underlying data can be found in S2 Data. FSI-FRET, Fully Quantified Spectral Imaging–Förster Resonance Energy Transfer; gH/gL, glycoproteins H and L; LBD, ligand-binding domain; WT, wild-type. (PDF) [file pbio.3001392.s010.pdf]
