# Supplementary material for: Human herpesvirus 8 molecular mimicry of ephrin ligands facilitates cell entry and triggers EphA2 signaling
Source: PLoS Biol. 2021 Sep 9;19(9):e3001392. doi: 10.1371/journal.pbio.3001392 (PMC8454987; doi:10.1371/journal.pbio.3001392)
Supplement: S1 Table — (DOCX) [file pbio.3001392.s015.docx]

# S1 Table: Crystallographic statistics

|  | | **HHV-8 gH/gL – EphA2 LBD** |
| --- | --- | --- |
| **Protein Data Bank code** | | 7B7N |
| **Crystallization conditions** | |  |
| Protein conc. (mg/ml) | | 5.1 |
| Crystallization buffer | 0.1M Na-malonate pH 5, 14.2% PEG 3350, 14 mM adenosine-5'-triphosphate disodium salt hydrate | |
| Crystallization method | | Sitting drop at 18°C |
| Cryo-protectant | | 20% ethylene glycol |
| **Data Collection^‡^** | |  |
| Beamline | | SOLEIL, Proxima 1 |
| Detector | | Eiger X 16M |
| Space group | | *C*222_1_ |
| Unit cell: a, b, c (Å) | | 72.94, 129.00, 267.69 |
| α, β, γ (°) | | 90, 90, 90 |
| Resolution (Å) | | 46.06-2.69 (2.79-2.69) |
| Measured reflections | | 479885 (44934) |
| Unique reflections | | 35310 (3328) |
| Completeness (%) | | 99.54 (96.24) |
| CC_1/2_ (%)* | | 99.7 (57.9) |
| Mean I/σ(I) | | 9.13 (0.87) |
| Multiplicity | | 13.6 (13.5) |
| B Wilson (Å^2^) | | 76.73 |
| Rsym | | 0.2387 (2.117) |
| Rmeas | | 0.2481 (2.199) |
| Rpim | | 0.06717 (0.5884) |
| **Structure Determination** | |  |
| MR search models | | EphA2 (PDB: 3HEI), EBV gH/gL (PDB: 3PHF) |
| N° of molecules in AU | | 1 gH/gL-EphA2 LBD complex |
| **Refinement^‡^** | |  |
| Resolution cut-off (Å) | | 46.06-2.69 (2.79-2.69) |
| Rwork (%) / Rfree (%) | | 21.6 / 24.2 (21.1 / 25.4) |
| N° of Work / Free reflections | | 33519 / 1762 (3161 / 165) |
| <B> atomic factors (Å^2^) | | 76.67 |
| N° of protein atoms | | 7082 |
| N° of solvent atoms/ions | | 54 / 98 |
| rmsd from ideal: | |  |
| Bond lengths (Å) | | 0.003 |
| Bond angles (°) | | 0.55 |
| Ramachandran^\|\|^ | |  |
| Favored (%) | | 95.47 |
| Allowed (%) | | 4.08 |
| Outliers (%) | | 0.45 |

^‡^Highest resolution shell is shown in parenthesis

*CC_1/2_ is the correlation coefficient (1)

^||^Ramachandran values from MolProbity (2)

# References

1. Karplus PA, Diederichs K. Assessing and maximizing data quality in macromolecular crystallography. Curr Opin Struct Biol. 2015;34:60-8.

2. Chen VB, Arendall WB, 3rd, Headd JJ, Keedy DA, Immormino RM, Kapral GJ, et al. MolProbity: all-atom structure validation for macromolecular crystallography. Acta Crystallogr D Biol Crystallogr. 2010;66(Pt 1):12-21.
