# Supplementary material for: Human herpesvirus 8 molecular mimicry of ephrin ligands facilitates cell entry and triggers EphA2 signaling
Source: PLoS Biol. 2021 Sep 9;19(9):e3001392. doi: 10.1371/journal.pbio.3001392 (PMC8454987; doi:10.1371/journal.pbio.3001392)
Supplement: S2 Table — The total surface area and the area at the interface, along with the number of atoms (Nat) and residues (Nres) are indicated for each pair of molecules. ΔG corresponds to the solvation free energy gain upon formation of the interface. Hydrogen bond distances cutoff of 3.5 Å, and 4.0 Å for salt bridges was applied; the number of hydrogen bonds and salt bridges are indicted with NHB and NSB, respectively. Residues forming salt bridges are indicated with bold letters. The contacts made by residue E52gH are shown in blue. The interface analyses were done in PDBePISA [9]. (DOCX) [file pbio.3001392.s016.docx]

# S2 Table: Interfaces between gH, gL and EphA2 LBD

The total surface area and the area at the interface, along with the number of atoms (N_at_) and residues (N_res_) are indicated for each pair of molecules. ΔG corresponds to the solvation free energy gain upon formation of the interface. Hydrogen bond distances cut-off of 3.5 Å, and 4.0 Å for salt bridges was applied; the number of hydrogen bonds and salt bridges are indicted with N_HB_ and N_SB_, respectively. Residues forming salt bridges are indicated with bold letters. The contacts made by residue E52^gH^ are shown in blue. The interface analyses were done in PDBePISA (1).

## A) gL interface with EphA2 LBD

| gL Surface  (Å^2^) | Interface area (Å^2^) | N_at_ | N_res_ | ΔG (kcal/mol) | N_HB_ | N_SB_ |
| --- | --- | --- | --- | --- | --- | --- |
| 7554 | 834 | 77 | 22 | -8.8 | 14 | 0 |

| **Inter-chain contacts** | | | |
| --- | --- | --- | --- |
| ## | gL | Dist. [Å] | EphA2 |
| 1 | L:ALA  31[ N  ] | 3.47 | E:ASP  61[ OD1] |
| 2 | L:SER  32[ N  ] | 3.18 | E:ASP  61[ OD1] |
| 3 | L:SER  32[ OG ] | 2.95 | E:ASP  61[ OD1] |
| 4 | L:ARG  63[ NH2] | 3.71 | E:MET  55[ SD ] |
| 5 | L:THR  70[ N] | 3.00 | E:LEU  54[ O  ] |
| 6 | L:GLU  72[ N] | 3.01 | E:GLN  56[ O  ] |
| 7 | L:VAL  22[ O  ] | 2.84 | E:ARG 103[ NH1] |
| 8 | L:VAL  22[ O] | 3.81 | E:CYS 188[ SG ] |
| 9 | L:GLN  30[ OE1] | 3.09 | E:ASN  60[ N ] |
| 10 | L:GLN  30[ OE1] | 3.13 | E:ASP  61[ N ] |
| 11 | L:ASP  68[ OD1] | 3.37 | E:TYR  48[ OH ] |
| 12 | L:THR  70[ O] | 3.01 | E:GLN  56[ N  ] |
| 13 | L:GLU  72[ OE1] | 3.03 | E:ASN  57[ ND2] |
| 14 | L:ASN 128[ OD1] | 3.83 | E:ASN  60[ N  ] |

## B) gH interface with EphA2 LBD

| gH Surface  (Å^2^) | Interface area (Å^2^) | N_at_ | N_res_ | ΔG (kcal/mol) | N_HB_ | N_SB_ |
| --- | --- | --- | --- | --- | --- | --- |
| 29843 | 121.4 | 15 | 4 | -0.4 | 2 | 1 |

| **Inter-chain contacts** | | | |
| --- | --- | --- | --- |
| ## | gH | Dist. [Å] | EphA2 |
| 1 | H:GLU  52[ O  ] | 2.91 | E:ARG  103[ NH2] |
| **2** | **H:GLU  52[ OE1]** | **2.94** | **E:ARG  103[ NH1]** |

## C) gL interface with gH

| gL Surface  (Å^2^) | Interface area (Å^2^) | N_at_ | N_res_ | ΔG (kcal/mol) | N_HB_ | N_SB_ |
| --- | --- | --- | --- | --- | --- | --- |
| 7554 | 2385 | 264 | 63 | -36.3 | 24 | 3 |

| **Inter-chain contacts** | | | |
| --- | --- | --- | --- |
| ## | gL | Dist. [Å] | gH |
| 1 | L:ILE  46[ N  ] | 2.90 | H:SER  48[ O  ] |
| 2 | L:PHE  48[ N  ] | 2.91 | H:GLU  50[ O  ] |
| 3 | L:VAL  22[ N  ] | 3.01 | H:GLU  52[ OE1] |
| 4 | L:HIS  47[ ND1] | 2.56 | H:GLU  52[ OE2] |
| 5 | L:ASN  79[ ND2] | 3.49 | H:ALA  81[ O  ] |
| 6 | L:ASN  79[ ND2] | 2.69 | H:GLU  82[ O  ] |
| 7 | L:ASN  76[ N  ] | 2.48 | H:VAL  83[ O  ] |
| 8 | L:ASN  76[ ND2] | 3.00 | H:GLU  85[ O  ] |
| 9 | L:ASN  79[ ND2] | 3.18 | H:GLU  85[ OE2] |
| 10 | L:ASN  76[ ND2] | 3.00 | H:THR  90[ OG1] |
| 11 | L:SER  82[ OG ] | 2.66 | H:TYR 172[ OH ] |
| 12 | L:ARG  90[ NH1] | 3.03 | H:PRO 173[ O  ] |
| 13 | L:ARG  89[ NH2] | 2.89 | H:ASP 227[ O  ] |
| 14 | L:ARG  89[ NH2] | 2.68 | H:LEU 229[ O  ] |
| 15 | L:ARG  89[ NH1] | 3.54 | H:SER 231[ OG ] |
| 16 | L:PRO  38[ O  ] | 2.99 | H:TRP  78[ NE1] |
| 17 | L:PHE  41[ O  ] | 2.76 | H:ARG  44[ NH2] |
| 18 | L:VAL  43[ O  ] | 2.88 | H:ARG  44[ NH1] |
| 19 | L:HIS  44[ O  ] | 3.55 | H:SER  48[ OG ] |
| 20 | L:HIS  44[ O  ] | 3.68 | H:SER  48[ N  ] |
| 21 | L:ILE  46[ O  ] | 2.65 | H:GLU  50[ N  ] |
| 22 | L:PHE  48[ O  ] | 2.95 | H:GLU  52[ N  ] |
| 23 | L:ASN  79[ O  ] | 3.36 | H:TYR 172[ OH ] |
| 24 | L:ASP 123[ OD1] | 3.67 | H:ARG  88[ NH2] |
| **25** | **L:HIS  47[ NE2]** | **3.67** | **H:GLU  50[ OE1]** |
| **26** | **L:HIS  47[ ND1]** | **2.56** | **H:GLU  52[ OE2]** |
| **27** | **L:ASP 123[ OD1]** | **3.67** | **H:ARG  88[ NH2]** |

# References

1. Krissinel E, Henrick K. Inference of macromolecular assemblies from crystalline state. J Mol Biol. 2007;372(3):774-97.
