# Supplementary material for: Human herpesvirus 8 molecular mimicry of ephrin ligands facilitates cell entry and triggers EphA2 signaling
Source: PLoS Biol. 2021 Sep 9;19(9):e3001392. doi: 10.1371/journal.pbio.3001392 (PMC8454987; doi:10.1371/journal.pbio.3001392)
Supplement: S3 Table — The total surface area and the area at the interface, along with the number of atoms (Nat) and residues (Nres) are indicated for the DIN in EphA2—free or bound to ligands. ΔG corresponds to the solvation free energy gain upon formation of the interface. The number of hydrogen bonds and salt bridges are indicted with NHB and NSB, respectively. The interface analyses were done in PDBePISA [9]. (DOCX) [file pbio.3001392.s017.docx]

# S3 Table: Properties of the dimerization interface (DIN) in EphA2 in unliganded form and bound to ligands

|  | Surface  (Å^2^) | Interface area (Å^2^) | N_at_ | N_res_ | ΔG (kcal/mol) | N_HB_ | N_SB_ |
| --- | --- | --- | --- | --- | --- | --- | --- |
| EphA2 ecto  PDB: 3FL7 | 25735 | 424 | 51 | 16 | -3.6 | 8 | 0 |
| EphA2 ecto + ephrin-A5  PDB: 2X11 | 26737 | 532 | 61 | 17 | -5.3 | 6 | 0 |
| EphA2 LBD-CRD + ephrin-A1  PDB: 3MBW | 15500 | 537 | 61 | 19 | -4.2 | 10 | 0 |
| EphA2 LBD + ephrin A1  PDB: 3CZU | 9301 | 634 | 70 | 17 | -6.9 | 7 | 0 |
| EphA2 LBD + HHV8 gH/gL  PDB: 7B7N | 8381 | 693 | 83 | 21 | -4.9 | 16 | 0 |

The total surface area and the area at the interface, along with the number of atoms (N_at_) and residues (N_res_) are indicated for the DIN in EphA2 - free or bound to ligands. ΔG corresponds to the solvation free energy gain upon formation of the interface. The number of hydrogen bonds and salt bridges are indicted with N_HB_ and N_SB_, respectively. The interface analyses were done in PDBePISA (1).

# References

1. Krissinel E, Henrick K. Inference of macromolecular assemblies from crystalline state. J Mol Biol. 2007;372(3):774-97.
