# Supplementary material for: Human herpesvirus 8 molecular mimicry of ephrin ligands facilitates cell entry and triggers EphA2 signaling
Source: PLoS Biol. 2021 Sep 9;19(9):e3001392. doi: 10.1371/journal.pbio.3001392 (PMC8454987; doi:10.1371/journal.pbio.3001392)
Supplement: S1 Text — (DOCX) [file pbio.3001392.s018.docx]

# S1 Text

# Supporting information: Material and methods

## HHV-8 gH/gL design of constructs for expression in insect cells and protein purification

The segments coding the ectodomain of HHV8 gH (residues 26 to 704) and the entire gL (residues 21 to 167) were cloned into the pT350 vector (1) for expression in insect S2 *Drosophila* cells. The Cys58 on gL was predicted to be unpaired based on the sequence alignment with EBV gL and was mutated to Ser to prevent potential formation of disulphide-linked dimers. The pT350 vector contains inducible metallothionein promoter activated by divalent cations, the exogenous *Drosophila* Bip signal peptide (MKLCILLAVVAFVGLSLG *RS*), underlined, that drives protein secretion appended to the N-terminus of the protein upstream of the 5’ cloning site. The *RS* are vector residues that code for the BglII cloning site. A double strep tag (DST) for affinity purification at the C-terminus was added downstream of the 3’ cloning site. The sequence of the DST in our pT350 plasmid is FEDDDDK*AG***WSHPQFEK***GGGSGGGSGGGS***WSHPQFEK**, where DDDDK is the enterokinase cleavage site and the sequences in bold correspond to the two strep tags separated by a GS linker (italics). The residues FE come from the vector *BstBI* cloning site.

HHV-8 gH can be secreted without being bound to gL (2), and the complex purification via a gH tag results in a mixture of the gH/gL complex and free gH, which are difficult to separate on SEC due to the small size difference (gH has a molecular weight of 75 kDa, and gL 20 kDa). This is why it was crucial for gH/gL purification to add the DST tag for affinity purification only on gL (gL^st^ where ‘st’ stands for strep tag, and gH^nt^ where ‘nt’ stands for no tag) (Fig 1A).

It is worth noting that due to the design of the pT350 vector for cloning via restriction digestion the expressed proteins end up having two extra residues RS (*BglII* site) at the N-terminus. The gLC58S^st^ pT350 and gH^nt^pT350 both contain the RS before the first authentic residues of the mature gL (Y21) and gH (L26).

The gH^nt^pT350 and gLC58S^st^pT350 plasmids were co-transfected in the S2 cells along with the plasmid encoding puromycin resistance, and the stably transfected cell lines were established by puromycin selection during 3-4 weeks following the previously established protocol (3). The expression was induced by addition of 0.5mM CuSO_4_, and the protein was purified from the supernatant 7-10 days post induction. After purification on Streptactin column (IBA Biosciences) and Superdex S200 16/60 GL column (GE life sciences), around 1 milligram of the gH/gL heterodimer was obtained per liter of cell culture (the same amount of the aggregated protein was present). SEC running buffer was 10 mM Tris, 50mM NaCl pH 8.0.

The reason mammalian cells were not used for production of the proteins used for crystallizations is that gH/gL complex is heavily glycosylated and the complex sugars added by mammalian cells are typically detrimental for protein crystallization, and cannot be enzymatically removed unlike the simple Endo-D and Endo-H sensitive carbohydrates added by insect cells.

## Preparation and purification of the trimeric complex EphA2 LBD-HHV8 gH/gL for crystallization

HHV-8 gH has 14 predicted N-glycosylation sites, gL one and EphA2 LBD none. To increase the probability that the complex would crystallize, the gH/gL complex was enzymatically deglycosylated with recombinant Endoglycosydase D (endo-β-N-acetylglucosaminidase from *Streptococcus pneumoniae* i.e. EndoD (4)), in 100mM sodium-citrate buffer pH 5.0, 150mM NaCl for 18h at 25°C. The ratio of protein to EndoD was 40 to 1 (w:w). To remove the EndoD and exchange the reaction buffer, the deglycosylated complex was purified on Superdex S200 column in 10 mM Tris, 50mM NaCl pH 8.0, and then mixed with the purified EphA2 LBD in 1:1.3 molar ratio (gH/gL : EphA2 LBD). The complex was incubated at 4°C for 1h to over-night, and then re-purified on Superdex S200 to separate the excess EphA2 LBD. The running buffer in all SEC purifications was 10mM Tris pH 8, 50mM NaCl. The trimeric complex was stable on SEC and presence of gH/gL and the EphA2 LBD in the complex peak was verified by SDS-PAGE gel analysis.

## The HHV-8 gH/gL-EphA2 LBD complex crystallization

The tertiary complex in 10 mM Tris pH 8, 50 mM NaCl was concentrated to 5.1 mg/ml in Vivaspin concentrators with the 10 kDa cut-off. Crystallization screening was performed at the Institut Pasteur Core facility for crystallization by vapor diffusion, in sitting drops of 0.4 μl containing equal volumes of the protein and reservoir solution (5). The drops were dispensed in 96-well Greiner plates by a Mosquito robot (TTPLabtech, Melbourn, UK) and images were recorder by a Rock-Imager 1000 (Formulatrix, Bedford, MA, USA). Initially, crystals were found in numerous conditions, but none diffracted better than 5Å. To improve these crystals, Hampton additive screen HT (HR2-138) was set up next based an initial hit, and crystals grown in 0.1M Na-malonate pH 5, 14.2% PEG 3350 in the presence of 14 mM adenosine-5’-triphosphate disodium salt hydrate diffracted to 2.7 Å.

## Data collection and structure determination

Data collected at the Proxima 1 beamline at the French national synchrotron facility (SOLEIL, St Aubin, France) were indexed, integrated, scaled and merged using XDS (6) and AIMLESS (7). Molecular replacement was done with Phaser within Phenix (8) using as search models the EphA2 LBD structure (PDB accession number 3HEI) and the HHV8 gH/gL model that was generated in Phyre2 (9) based on the sequence similarity with the EBV gH/gL (PDB code 3PHF). The partial solution containing EphA2 LBD and parts of gH was obtained, and was extended by iterative rounds of model building (Autobuild (10) in Phenix and manual building in Coot (11)) and refinement using Buster (12) and Phenix (13). The final model converged to R_work_/R_free_ of 0.22/0.24. The final map displayed clear electron density for residues 27-200 of EphA2 (with a break in the J helix region (residues 148-162) and GH loop (G111)), residues 21-128 for gL, and residues 35-696 gH with the exception of several short regions of poor density (breaks, ‘*b’*) that precluded unambiguous placement of the polypeptide chain as indicated in Fig 1A : *b^1^* (111), *b^2^* (148-162) in EphA2 LBD, and *b^3^*(127-131), *b^4^*(212-216), *b^5^*(521-526), *b^6^*(547-550), *b^7^*(558-559), *b^8^*(627-629) in gH.

The crystallization conditions, crystal parameters, data statistics, and refinement parameters are shown in Table S1. Superpositions of structures and all structural figures were generated with PyMOL (version 1.3r1) (14). The atomic coordinates and structure factors for trimeric complex EphA2 LBD-HHV8 gH/gL have been deposited in the RCSB Protein Data Bank with the PDB code 7B7N.

## HHV-8 gH/gL: design of constructs for transient expression in mammalian cells

The mammalian cell expression constructs for gH and gL (residues 26-704 and 21 to 167, respectively) contained at the N-termini an exogenous murine Ig κ-chain leader sequence that targets protein to secretory pathway (METDTLLLWVLLLWVPGSTG) (15). Different affinity tags were added to their C-termini - for gH construct (gH^his^pcDNA3.1) an enterokinase cleavage site (underlined) flanked by two GS linkers (italics) was followed by an octa-his tag (bold) (*GS* DDDDK *SGS* **HHHHHHHH**), and for gL a DST (gL^st^pcDNA3.1; the DST sequence is the same as in the gLC58S^st^pT350 construct described above). The EphA2 variants contained the endogenous signal peptide at the N-terminus and ended at residue 534 followed by a DST tag. Proteins were expressed by transient transfection of Expi293 cells, using 293Expifectamine reagent for transfection, following the manufacturer’s protocol (Thermo Fisher Scientific). Proteins were purified from the supernatants 5-7 days after transfection by affinity chromatography using Streptactin columns (IBA Bioscience, for proteins containing DST) or HisTrapExCel columns (Cytiva, for proteins containing his tags), followed by SEC purification.

## Complex formation for SEC-MALS measurements

The complexes were formed by incubating gH^his^/gL^st^ and EphA2^st^, both produced in mammalian cells as described above, in 1:1.3 molar ratio for 30 minutes at 4°C (∼100 μg of gH/gL were mixed with 80 μg of EphA2 ectodomains or 30 μg of the EphA2 LBD in PBS in total volume of 200 μl). Assembled complexes were injected into Superdex 200 10/300 GL column (GE life sciences) using a 500 μl loop, and run in PBS at a flow rate of 0.4 ml/min.

## Cell culture used in FRET and contraction experiments

Human embryonic kidney (HEK) 293T cells were purchased from American Type Culture Collection (Manassas, VA; CRL-3216). The cells were cultured at 37 °C and 5% CO_2_ in Dulbecco’s Modified Eagle Medium (DMEM; Thermo Scientific; 31600-034) that contained 3.5 g/L D-glucose, 1.5 g/L sodium bicarbonate, and 10% fetal bovine serum (FBS; Sigma-Aldrich; F4135). The cells were passed up to 25 times and then discarded.

## FSI-FRET measurements and analysis

For FRET experiments, the cells were seeded in 35 mm glass bottom collagen-coated petri dishes (MatTek Corporation, MA) at a density of 2 x 10^5^ cells/dish and cultured for 24 hours. The cells were co-transfected with EphA2-mTurquoise (mTURQ, the donor) and EphA2-enhanced yellow fluorescent protein (eYFP, the acceptor) in pcDNA, as described (16), using 0.5-2 μg of total DNA and the Lipofectamine 3000 reagent (Invitrogen, CA). In control experiments, cells were transfected with either EphA2-mTURQ or EphA2-eYFP and used for calibration as described (17). Twelve hours following transfection, the cells were washed twice with serum-free, phenol red-free media and serum-starved in the same media for 12 hours overnight. Immediately before imaging, the starvation media was replaced with hypo-osmotic media (10% starvation media, 90% diH_2_O, 25 mM HEPES) to ‘unwrinkle’ the highly ruffled cell membrane under reversible conditions as described (18). The soluble proteins were premixed with the hypo-osmotic media before adding to the cells. Cells were incubated for 10 minutes and then imaged under these conditions for approximately 1 hour.

Spectral images of HEK293T cells under reversible osmotic conditions were obtained following published protocols (17) with a spectrally resolved two-photon microscope (Zeiss Inverted Axio Observer) equipped with line-scanning capabilities (OptiMis True Line Spectral Imaging system, Aurora Spectral Technologies, WI) (19). Fluorophores were excited by a mode-locked laser (MaiTai™, Spectra-Physics, Santa Clara, CA) that generates femtosecond pulses between wavelengths 690 nm to 1040 nm. Two images were collected for each cell: one at 840 nm to excite the donor and a second one at 960 nm to primarily excite the acceptor. Solutions of purified soluble fluorescent proteins (mTURQ and eYFP) at known concentrations were produced following a published protocol (20) and imaged at each of these excitation wavelengths. A linear fit generated from the pixel-level intensities of the solution standards was used to calibrate the effective three-dimensional protein concentration which can be converted into two-dimensional membrane protein concentrations in the cell membrane as described (17). The calibration curve along with the cell images were used to calculate the FRET efficiency and the concentration of donors and acceptors present in the cell membrane (17). Regions of the cell membrane not in contact with neighboring cells were selected and analyzed to avoid interactions with proteins on adjacent cells.

The measured FRET efficiencies (*E_app_*) were corrected for ‘proximity FRET’ (*E_prox_*) as described previously (21). The proximity FRET accounts for donor-tagged molecules and acceptor-tagged molecules coming into close enough proximity to observe a FRET signal (within 100 Å) but not interacting directly. The corrected FRET due to specific interactions between the labeled proteins is given by:

$FRET=\frac{E_{prox}-E_{app}}{{2E}_{prox}-E_{prox}E_{app}-1}$ (1)

The corrected FRET depends on the fraction of membrane protein dimers, *f_D_*, and on the acceptor fraction, *x_A_*, according to:

$FRET=f_{D}x_{A}\tilde{E}$ (2)

The ‘Intrinsic FRET’ (Ẽ) is a structural parameter that depends on the distance between and orientation of the two fluorophores in the dimer but is not dependent on the dimerization propensity (21-23).

When the dimeric fraction is 100% (*f_D_* = 1), the corrected FRET does not depend on the concentration of the labeled proteins and thus equation (2) can be simplified further:

$FRET=x_{A}\tilde{E}.$ (3)

The dependence of the Intrinsic FRET on the distance between the fluorescent proteins in the dimer, *d*, is given by (22):

$Ẽ=\frac{1}{1+\left( \frac{d}{R_{0}} \right)^{6}}$ (4)

where *R_0_* is the Forster radius for the mTurquoise-eYFP FRET pair, 54.5 Å. Since the fluorescent proteins are attached to the C-terminus of the membrane proteins via long flexible linkers, we assume free rotation of the fluorescent proteins.

By rearranging equation (2), the dimeric fraction, *f_D_*, can be determined from the corrected FRET efficiencies and concentrations according to:

$f_{D}\boldsymbol{=} \frac{FRET}{x_{A}Ẽ}$ (5)

In the case of dimers, the following equation is used to determine the two unknowns *K_diss_* and Ẽ as described previously (17):

${\frac{FRET}{x_{A}}}=\frac{1}{\left[ R_{total} \right]}([R_{total}]-\frac{K_{diss}}{4}\left( \sqrt{1+8\left[ R_{total} \right]/K_{diss}}-1 \right)\tilde{E}$ (6)

## Fluorescence intensity fluctuations (FIF) spectroscopy measurements and analysis

HEK293T cells were seeded as described in the FRET section at a density of 4 x 10^5^ cells/dish. The cells were transiently transfected 24 hours later with 1 μg of EphA2-eYFP using Lipofectamine 3000 and then washed and serum starved twelve hours later. The starvation media was replaced with a 75% hypo-osmotic media (25% starvation media, 75% diH2O, 25 mM HEPES) containing 200 nM gH/gL prior to imaging. A TCS SP8 confocal microscope (Leica) using the photon counting capabilities of the HyD hybrid detector was used to collect images of the cell basolateral membranes. The measurements were performed with a 488 nm excitation diode laser and the emission spectra of YFP were collected from 520-580 nm. The scanning speed was at 20Hz, the pixel depth at 12-bits, the zoom factor at 2, and the image size at 1024x1024.

The cell images were analyzed using the FIF software described in (24). The software performed segmentation of the basolateral membrane into 15x15 pixel regions of interest. Each cell is outlined by researcher prior to segmentation. The segmented data was analyzed using the brightness and concentration calculator in the FIF software (24). The molecular brightness, ε, was calculated according to:

$\varepsilon=\frac{\sigma^{2}-\sigma_{D}^{2}}{<I>}$ (7)

where $\sigma^{2}$ is the variance of fluorescence across segments, $\sigma_{D}^{2}$ is the variance of the noise of the detector, and $<I>$ is the average fluorescence intensity, for a photon-counting detector as used here, the brightness is (25, 26):

$\varepsilon=\frac{\sigma^{2}}{<I>}-1$ (8)

The brightness values, calculated for thousands of regions of interest, were potted as histograms.

# References

1. Krey T, d'Alayer J, Kikuti CM, Saulnier A, Damier-Piolle L, Petitpas I, et al. The disulfide bonds in glycoprotein E2 of hepatitis C virus reveal the tertiary organization of the molecule. PLoS Pathog. 2010;6(2):e1000762.

2. Hahn A, Birkmann A, Wies E, Dorer D, Mahr K, Sturzl M, et al. Kaposi's sarcoma-associated herpesvirus gH/gL: glycoprotein export and interaction with cellular receptors. J Virol. 2009;83(1):396-407.

3. Backovic M, Krey T. Stable Drosophila Cell Lines: An Alternative Approach to Exogenous Protein Expression. Methods Mol Biol. 2016;1350:349-58.

4. Fan SQ, Huang W, Wang LX. Remarkable transglycosylation activity of glycosynthase mutants of endo-D, an endo-beta-N-acetylglucosaminidase from Streptococcus pneumoniae. J Biol Chem. 2012;287(14):11272-81.

5. Weber P, Pissis C, Navaza R, Mechaly AE, Saul F, Alzari PM, et al. High-Throughput Crystallization Pipeline at the Crystallography Core Facility of the Institut Pasteur. Molecules. 2019;24(24).

6. Kabsch W. XDS. Acta Crystallogr D Biol Crystallogr. 2010;66(Pt 2):125-32.

7. The CCP4 suite: programs for protein crystallography. Acta Crystallogr D Biol Crystallogr. 1994;50(Pt 5):760-3.

8. Adams PD, Afonine PV, Bunkoczi G, Chen VB, Davis IW, Echols N, et al. PHENIX: a comprehensive Python-based system for macromolecular structure solution. Acta Crystallogr D Biol Crystallogr. 2010;66(Pt 2):213-21.

9. Kelley LA, Mezulis S, Yates CM, Wass MN, Sternberg MJ. The Phyre2 web portal for protein modeling, prediction and analysis. Nat Protoc. 2015;10(6):845-58.

10. Terwilliger TC, Grosse-Kunstleve RW, Afonine PV, Moriarty NW, Zwart PH, Hung LW, et al. Iterative model building, structure refinement and density modification with the PHENIX AutoBuild wizard. Acta Crystallogr D Biol Crystallogr. 2008;64(Pt 1):61-9.

11. Emsley P, Cowtan K. Coot: model-building tools for molecular graphics. Acta Crystallogr D Biol Crystallogr. 2004;60(Pt 12 Pt 1):2126-32.

12. Bricogne G, Blanc E, Brandl M, Flensburg C, Keller P, Paciorek W, et al. BUSTER. 2.8.0 ed. Cambridge, United Kingdom: Global Phasing Ltd.; 2009.

13. Liebschner D, Afonine PV, Baker ML, Bunkoczi G, Chen VB, Croll TI, et al. Macromolecular structure determination using X-rays, neutrons and electrons: recent developments in Phenix. Acta Crystallogr D Struct Biol. 2019;75(Pt 10):861-77.

14. DeLano WL. The PyMOL Molecular Graphics System. San Carlos, CA, USA: DeLano Scientific; 2002.

15. Coloma MJ, Hastings A, Wims LA, Morrison SL. Novel vectors for the expression of antibody molecules using variable regions generated by polymerase chain reaction. J Immunol Methods. 1992;152(1):89-104.

16. Singh DR, Kanvinde P, King C, Pasquale EB, Hristova K. The EphA2 receptor is activated through induction of distinct, ligand-dependent oligomeric structures. Commun Biol. 2018;1:15.

17. King C, Stoneman M, Raicu V, Hristova K. Fully quantified spectral imaging reveals in vivo membrane protein interactions. Integr Biol (Camb). 2016;8(2):216-29.

18. Sinha B, Koster D, Ruez R, Gonnord P, Bastiani M, Abankwa D, et al. Cells respond to mechanical stress by rapid disassembly of caveolae. Cell. 2011;144(3):402-13.

19. Biener G, Stoneman MR, Acbas G, Holz JD, Orlova M, Komarova L, et al. Development and experimental testing of an optical micro-spectroscopic technique incorporating true line-scan excitation. Int J Mol Sci. 2013;15(1):261-76.

20. Sarabipour S, King C, Hristova K. Uninduced high-yield bacterial expression of fluorescent proteins. Anal Biochem. 2014;449:155-7.

21. King C, Raicu V, Hristova K. Understanding the FRET Signatures of Interacting Membrane Proteins. J Biol Chem. 2017;292(13):5291-310.

22. Chen L, Novicky L, Merzlyakov M, Hristov T, Hristova K. Measuring the energetics of membrane protein dimerization in mammalian membranes. J Am Chem Soc. 2010;132(10):3628-35.

23. Sarabipour S, Del Piccolo N, Hristova K. Characterization of membrane protein interactions in plasma membrane derived vesicles with quantitative imaging Forster resonance energy transfer. Acc Chem Res. 2015;48(8):2262-9.

24. Stoneman MR, Biener G, Ward RJ, Pediani JD, Badu D, Eis A, et al. A general method to quantify ligand-driven oligomerization from fluorescence-based images. Nat Methods. 2019;16(6):493-6.

25. Ahmed F, Zapata-Mercado E, Rahman S, Hristova K. The Biased Ligands NGF and NT-3 Differentially Stabilize Trk-A Dimers. Biophys J. 2021;120(1):55-63.

26. Fox M. Quantum optics: an introduction: Oxford Univeristy Press; 2006.
