# Supplementary material for: Human herpesvirus 8 molecular mimicry of ephrin ligands facilitates cell entry and triggers EphA2 signaling
Source: PLoS Biol. 2021 Sep 9;19(9):e3001392. doi: 10.1371/journal.pbio.3001392 (PMC8454987; doi:10.1371/journal.pbio.3001392)
Supplement: S2 Text — (DOCX) [file pbio.3001392.s019.docx]

# References for SI (Figure captions for S1-S12, S1-S3 Tables)

1. Singh DR, Kanvinde P, King C, Pasquale EB, Hristova K. The EphA2 receptor is activated through induction of distinct, ligand-dependent oligomeric structures. Commun Biol. 2018;1:15.

2. Gomez-Soler M, Gehring MP, Lechtenberg BC, Zapata-Mercado E, Hristova K, Pasquale EB. Engineering nanomolar peptide ligands that differentially modulate EphA2 receptor signaling. Journal of Biological Chemistry. 2019;294(22):8791-805.

3. Himanen JP, Goldgur Y, Miao H, Myshkin E, Guo H, Buck M, et al. Ligand recognition by A-class Eph receptors: crystal structures of the EphA2 ligand-binding domain and the EphA2/ephrin-A1 complex. EMBO Rep. 2009;10(7):722-8.

4. Himanen JP, Rajashankar KR, Lackmann M, Cowan CA, Henkemeyer M, Nikolov DB. Crystal structure of an Eph receptor-ephrin complex. Nature. 2001;414(6866):933-8.

5. Su C, Wu L, Chai Y, Qi J, Tan S, Gao GF, et al. Molecular basis of EphA2 recognition by gHgL from gammaherpesviruses. Nat Commun. 2020;11(1):5964.

6. Holm L. DALI and the persistence of protein shape. Protein Sci. 2020;29(1):128-40.

7. Sievers F, Wilm A, Dineen D, Gibson TJ, Karplus K, Li W, et al. Fast, scalable generation of high-quality protein multiple sequence alignments using Clustal Omega. Mol Syst Biol. 2011;7:539.

8. Gouet P, Courcelle E, Stuart DI, Metoz F. ESPript: analysis of multiple sequence alignments in PostScript. Bioinformatics. 1999;15(4):305-8.

9. Krissinel E, Henrick K. Inference of macromolecular assemblies from crystalline state. J Mol Biol. 2007;372(3):774-97.
