# Supplementary material for: Human herpesvirus 8 molecular mimicry of ephrin ligands facilitates cell entry and triggers EphA2 signaling
Source: PLoS Biol. 2021 Sep 9;19(9):e3001392. doi: 10.1371/journal.pbio.3001392 (PMC8454987; doi:10.1371/journal.pbio.3001392)
Supplement: Attachment — Submitted filename: Response to Reviewers.pdf [file pbio.3001392.s021.pdf]

Letter from the Editor:

Dear Dr Backovic,

Thank you very much for submitting your manuscript "Human herpesvirus 8 molecular mimicry of ephrin ligands facilitates cell entry and triggers EphA2 signaling" for consideration as a Research Article at PLOS Biology. Please accept my apologies for the delay in getting back to you with our initial decision. Your manuscript has been evaluated by the PLOS Biology editors, an Academic Editor with relevant expertise, and by four independent reviewers.

The reviews are attached below. You will see that the reviewers find your conclusions novel and interesting, but ask that the Introduction and Discussion sections are shortened and that the gL/gH-EphA2 structure is directly compared with the previously reported structure in Su et al (Reference 43) to highlight their similarity. In addition, Reviewer #1 requests that additional methodological and technical reporting details for the FSI-FRET data are included in the manuscript. Please address all of the reviewers concerns.

As you suggested we have:

- Reorganized and shortened the Introduction and Discussion sections, which are now more succinct.
- Added the comparison with the structure of the same complex reported earlier by Su *et al.* (reference 34 in the revised manuscript) in the Results section (lines 148-154) and added a Figure (S3B Fig) to illustrate the few differences in the two structures (they are indeed quite similar).
- Restructured the discussion section to contain subsections, which we hope will make the contributions and novelty of our findings stand out better.
- Uploaded the correct version of the SI that contains the technical details related to the FSI-FRET experiments Reviewer #1 asked about.

Reviewer #1:

The authors investigated the interaction between HHV-8 envelope glycoprotein complex gH/gL and cellular EphA2 tyrosine kinase receptor using a few biochemical/biophysical tools in this manuscript. They revealed exciting features of gH/gL as mimicry of ephrins - the physiological receptor of EphA2. The authors performed FSI-FRET (Fully Quantified Spectral Imaging Fluorescence Resonance Energy Transfer) to characterize the association of EphA2 upon binding with gH/gL. They found that HHV-8 gH/gL induced EphA2 dimerization with a serial of EphA2 and gH/gL mutants as controls. From the FRET perspective, the results in Figs 4-5 and the original data in Fig. S8 are sounding but lacking methodologic details. Although the authors indicated that all the experimental information of FSI-FRET was in the SI, I did not find such information. I did not find the equation 1, 4, 6 associated with the quantification of FSI-FRET data.

We thank the reviewer and apologize for having submitted (by mistake) an older version of the SI that did not include this information. We have now updated the SI and all the methodological details and equations related to the FSI-FRET and FIF measurements that can be found on pages 6-8 of the revised SI.

Some minor suggestions:

1) It was not easy for me to find the essential details. The authors may consider constructing and condensing the content.

We agree that the previous version of the manuscript, especially the Introduction and Discussion, contained information that were not necessary for a reader to follow the findings we present. We have shortened the Introduction for about third, keeping only the relevant information, and restructured and trimmed the Discussion so that our results and conclusions stand out better.

2) In FSI-FRET in Fig.S8, the authors did not mention the number of experimental repeats. In the figure legend, It was not clear to me what the measured receptor concentration is. Is it the total concentration of EphA2-mTURQ and EphA2-eYFP or just EphA2-eYFP? My understanding is that EphA2-mTURQ is the donor-tagged, not the acceptor-tagged.

The FSI-FRET data in Fig. SI8 (now Fig S10) were combined from at least 10 experimental repeats. We have added this information to the figure legend.

We apologize that the receptor concentration was not clear from the figure legend. This information was provided in the Figure SI8 legend "*The FRET efficiencies were corrected for the non-specific 'proximity FRET' contribution and are plotted as a function of the measured receptor concentration (EphA2-mTURQ+EphA2-eYFP concentrations)*." We now include this information in the legend of the Figure 4 in the main text as well. The reviewer is also correct that EphA2-mTURQ is the donor molecule while EphA2-eYFP is the acceptor molecule. The distributions of the measured EphA2-mTURQ and EphA2-eYFP concentrations are provided in Fig. SI8 and both are included in the data fitting process.

3) Results of key FSI-FRET are in Figs.4-5, derived from Fig.S8. Does 'untreated' in several subplots mean the absence of the associated gH/gL? In Fig.5B-D, does ' EphA2 WT' stand for 'EphA2 WT + gH/gL' as shown in Fig.4? It would only make sense if it did. Otherwise, results are not consistent between Fig.4 and Fig.5.

The reviewer is correct, and we apologize for not making this clear. We now include the word "untreated" in the figure legends to be more consistent with the figure and to indicate that no gH/gL or other ligand is present. For Figures 4 and 5, we provided the information in the figure legends, but we have now also revised the figures to keep the labeling scheme consistent.

Reviewer #2:

Manuscript No.: PBIOLOGY-D21-01060R1

Title: Human herpesvirus 8 molecular mimicry of ephrin ligands facilitates cell entry and triggers EphA2 signaling

Authors: T. P. Light, D. Brun, P. Guardado-Calvo, R. Pederzoli, A. Haouz, F. Neipel, F. Rey, K. Hristova and M. Backovic

Entry of herpes viruses into a host cell is found to involve the four core or quartet of virus-encoded glycoproteins, gD, gB and the gH/gL complex. In the best-known pathway, gD attaches to the host cell surface getting gB close to the receptor, gB binds to the receptor and mediates fusion between the virus and cell membranes while gH/gL activates gB for the fusion process. This pathway is thought to be involved, for instance, in entry of the alpha-herpesviruses HSV1, HSV2, PRV and VZV.

The pathway is somewhat different in the case of the gamma-herpesviruses KSHV and EBV. Here the cell surface receptor for the virus has been identified and shown to be the ephrin receptor (EphA2), but the receptor is bound by the gH/gL complex rather than by gB. The gH/gL complex is suggested to be a molecular mimic of ephrin, the cellular ligand for EphA2.

In the present submission, the authors report a structural analysis by X-ray crystallography of the purified EphA2 receptor binding domain attached to gHgL. The results provide strong support for the idea that gH/gL binds EphA2 at the same site as the cellular ligand, ephrin (compare Figs. 3A and 3B). For instance, the gH residue E52 is found to be in the same site as ephrin E119. The authors complement their crystallographic analysis with biochemical studies of proteins with mutations in the amino acids suggested to be involved in EphA2-gHgL contacts. The work is very well done, thorough and described clearly in the manuscript.

The most important weakness of the paper has to do with the fact that a similar crystallographic analysis of both KSHV and EBV EphA2-gH/gL interactions has recently been published (see ref. 43). The prior paper appeared while the current manuscript was being prepared. The prior paper is of high quality and it reaches the same conclusions as the current submission. The Editor of PLoS Biology will need to make the decision

regarding whether to publish the present submission. I would emphasize the high quality and convincing conclusions reached in both studies.

We thank the reviewer for the comments and emphasizing the quality of our manuscript.

We have been aware of this issue all along, and decided to submit the manuscript to PLOS Biology because of its policy to "*waive the novelty criteria for work submitted within six months of a similar study having been published*". While the structures we and Su *et al.* have solved are indeed very similar, our work provides additional findings, which were not reported before:

- Mutagenesis studies targeting gH, gL and EphA2 that now provide a complete validation of the binding site
- We show that single gH residue (E52) that makes contacts with EphA2 is essential for high affinity interactions, explaining previous literature pointing to the importance of gH for binding to EphA2 (Grobkopf AK *et al.* (2018)).
- We provide additional key structural details revealed due to higher resolution of our structure (2.7Å vs 3.2Å reported by Su *et al.*)- this was especially informative when establishing the structural mimicry between gH/gL and ephrins
- We show that monomeric, and not dimerized or pre-clustered gH/gL can induce cell contraction
- Importantly, we demonstrate that gH/gL induced dimerization of EphA2 *in vivo* occurs via a specific EphA2 interface, a finding that may provide to the clues how to perturb EphA2 signaling

I feel the current submission would be enhanced by a graphical abstract illustrating the KSHV entry pathway suggested by the results reported. This would be of help to readers unfamiliar with the gamma-herpesvirus literature.

We appreciate this comment and completely agree. We have added the schematic representation of an HHV-8 virion and its entry pathway in S1 Fig.

The results of the current study and of ref 43 both suggest that it might be possible for KSHV and EBV to enter a host cell without involvement of gB. I believe this idea should be mentioned somewhere in the current manuscript.

This is an interesting remark, and we thank the reviewer for drawing our attention to this point. We agree that based solely on our and the findings by Su *et al.* one could infer that gH/gL binding to EphA2, which as a consequence increases endocytosis of the virus, might be sufficient to allow the virus to enter the cells. The situation is however more complex, and to our knowledge there haven't been any experimental evidence indicating that HHV-8 can enter cells without gB. It would be very interesting to try to block gB on the virus via antibodies or else, and test if such virus would be internalized at the same level as the WT virus. Here is a more detailed explanation of why gB is needed in addition to gH/gL for HHV-8 entry.

The capsids of herpesviruses that enter by fusion of the viral and *plasma membrane* of target cells (such as HSV-1, VZV, CMV) gain access to the cytosol immediately upon fusion, and entry and membrane fusion are concurrent processes. But the herpesviruses that are endocytosed, such as HHV-8, have the capsids released from the endosomal compartment. So the entry step in the latter case requires internalization of the virus (which maybe is not dependent on gB) and then membrane fusion in the endosomes, which requires gB. It is known that the fusion step in HHV-8 is mediated, as in all other herpesviruses, by the concerted action of fusogenic gB and gH/gL. So while HHV8 without gB could in theory be internalized by cells, it would remain stuck in the endosomes and would not be infectious as the capsids would not be released into the cytosol. The reason we do not know – to the best of our knowledge – if a HHV8 devoid of gB would be able to gain access to cells via endocytosis is that HHV8 gB is essential for virus egress. When HHV-8 gB was knocked out, all the viral particles were blocked inside the cell (reference) precluding generations of ΔgB HHV-8 virus that is needed to test the hypothesis.

The critical role of gB in HHV-8 entry is corroborated by cell-cell fusion assays that partially emulates fusion of the viral and cellular membranes. HHV-8 gH/gL by itself is not sufficient to mediate fusion, and gB has to be co-transfected (reference).

We have modified the text in the Introduction to clearly state the requirement of gB for HHV-8 entry (lines 51-55).

Reviewer #3:

The article by Light et al. describes the structure of the HHV-8 envelope glycoprotein gH/gL complex bound to the EphA2 tyrosine kinase receptor, and by exhaustive targeted mutagenesis using FSI-FRET, demonstrates that the gH/gL mimics the ephrins ligand binding mode to EphA2 and induces a similar cellular response by favoring the dimerization of the EphA2 and cell contraction.

Overall, the manuscript is a consistent piece of work and should be published in PLoS Biology after the minor suggested revisions are addressed.

A general comment is that the manuscript is over-long in both the Introduction and Discussion sections, and efforts should be made to be more concise. Further, it is worth noting that an equivalent structure was published by others in Nature Communication (Su et al., 2020) and the Authors here make an important effort to expand upon the implications of the solid structural results in the cellular context using in-cell FRET and contraction assay techniques.

Here follows a few suggestions that would possibly help in the clarity of the text.

INTRODUCTION

Pages 4-5, lines 103-141: This is a very detailed description of the Eph receptors' structure and biology - would it be possible to introduce such aspects more succinctly and refer the more curious reader to a review? (Surely this is possible)

We agree - the text in the introduction is shortened for around a third, with clear references to reviews where more information can be found. Some of the text was moved to S1 and S2 figure legends.

Page 6, line 155: Please avoid 'This is the first time…..' as the quality of results should awaken the interest and underscore the merits.

We agree and we have modified the text to avoid this statement.

RESULTS:

Page 7, lines 163-181: This paragraph should be moved to the Material and Methods section and merged with the paragraph 'Data collection and structure determination' on page 22 (lines 628-643).

The text has been modified as suggested.

Also, referring to the space group please use the format: C 2 2 21 , with the C in italics and the sub-index 1 across the entire text and supplementary information (Table S1 included).

Thank you for this remark, we completely agree and we have changed it.

The Figure 1A can be moved to the paragraph 'The gH/gL-EphA2 LBD complex structure'.

We have followed the suggestion and modified the text accordingly.

Page 9, lines 233-236: it would be beneficial to add in the SI the gel corresponding to the shift in migration between the native and N-linked mutated versions. Also, proteomics on the mutated versions would unequivocally confirm the presence of glycosylation on the mutated residues. Is there any reason why proteomics was not employed for this check?

The N-glycosylation mutants gL mutants (gL D30N and gL Q68N) showed a clear shift towards higher molecular weight when analyzed by SDS PAGE. We now presented this data (S7 Fig). The EphA2 glycosylation mutant A190N, L192S was already reported (PMID 20228801) and shown to be defective in accumulating at cell-cell contacts, indicating perturbed interactions with the ephrinA5 ligands due to the glycosylation. The reason we had not employed proteomics was that we could not get a fast / easy access to a proteomics facility during previous year, and since we had some evidence, described above, of the presence of glycosylations at the newly introduced sites we thought that the proteomics analysis was not essential.

Page 10, lines 282-285: How do the Authors know how many labelled EphA2 molecules are decorating the HEK cells? Why do the Authors use 200 nM of HHV-8 gH/gL and not 100 nM? How do the Authors know - a priori - that with 200 nM of HHV-8 gH/gL they are saturating all labelled EphA2 on the cells? Can the Author clarify this sentence and better contextualize it with Figure S8?

We apologize for having submitted an older version of the SI that did not include this information. The methodological details of the FSI-FRET measurements can be found on pages 6-7 of the revised SI. In brief, the concentration of EphA2-mTurq and EphA2-eYFP were determined by deconvoluting the pixel-level fluorescence spectra and converting the fluorescence intensities into 2D concentrations using a calibration curve generated by imaging solutions of purified fluorescent proteins at known concentration.

The 200 nM gH/gL concentration used for FRET, FIF, and contraction assay experiments was selected so that the concentration was sufficiently high to ensure binding to EphA2 given that the K_d is in the low nM range. The 100 nM concentration mentioned by the reviewer would have likely provided the same results but we chose 200 nM from the beginning and used it consistently.

200 nM is approximately 1.2×10^{13} gH/gL molecules given that the final volume of the solution containing the gH/gL is 1 mL. In our experiments, we seed each imaging dish with 2×10^5 cells. HEK293T cells have a doubling time of approximately 20 hours which would be approximately 1×10^6 cells after 48 hours at which point the cells are imaged. The cells when swollen have a radius of approximately 10 μm which is approximately 1,256 μm^2 surface area (assuming a sphere). The highest EphA2 expression we observe in our experiments is approximately 8,000 receptors/μm^2 and the lowest is approximately 100 receptors/μm^2. If we multiply the number of cells, the surface area of the cells, and the concentration of receptors (using the highest expression), we arrive at approximately 1×10^{13} EphA2 molecules. Therefore, we have an excess of gH/gL molecules compared to EphA2 molecules even at the highest receptor estimate.

Page 10, line 295: the references [64-65] do not follow the journal style, and, moreover they are not sequential to the previous reference 60 on page 8 line 228. Please check.

We fixed this overlooked error, thank you for drawing our attention to it.

Page 13, lines 395-399: It would be preferable to provide some sort of quantification to the expressions 'same extent' and 'cells were slightly smaller'. As they are - the sentences appear a bit vague. Can the Authors provide approx. %?

We appreciate the reviewer’s comment and have now added the approximate percent change in cell surface area and altered the text to be clearer.

DISCUSSION:

The Discussion section nicely unfolds but its length undermines the delivery of the take-home message. A more concise writing would help to focus the discussion and leave the reader with the essential analysis.

We agree and we have shortened and restructured Discussion into subsections to make the essential findings stand out.

Page 15, lines 423 and 426: 'charged residues', from the underlined residues it appears that these residues are positively charged residues - should this be made explicit?

We modified the text as suggested.

Page 16, lines 443-455: this paragraph should be re-phrased, first stating the Kd obtained via BLI and the design of the experiment, and then contextualized with the published literature. In particular the sentence 'We therefore think that our data represents the most accurate quantification…..' should be avoided, leaving the results and rigor of the methodology used for the task to highlight the merit of the findings.

We fully agree. The text has been modified accordingly.

FIGURES:

Figure 1: Panel A, the letter size of b, b' etc, and the yellow numbers are too small - please correct them to improve interpretability. Panel B, the pink asterisk is not clearly visible within the magenta EphA2 domain - please could you use a more contrasting color?

Also, in the gH protein there are residues as stick and colored in yellow which should be clarified as representing disulfide bonds (in particular as the same type of representation is used in Figure S4).

We introduced suggested modifications and edited the Fig 1 legend.

Figure 2: Panel A, the inset at the bottom right is too small to be discernible. Panel D is too small to be clearly interpretable.

We redid Figure 2 so that the insert in panel A and plots in panel D are discernable.

Reviewer #4:

Light et al provide a tour de force analysis of herpes virus HHV-8 gLgH glycoprotein with its cellular receptor EphA2 that normally functions as a tyrosine kinase (ephrin ligand) receptor. The authors show that the structure of the gLgH/EphA2 ligand binding domain complex closely resembles the mode of binding of the ephrin ligand to the EphA2 ligand binding domain and prepare and test a series of mutations to residues of the glycoprotein complex predicted to interrupt the interaction and they do. They then perform a series of experiments to demonstrate that binding of the glycoprotein complex to full length EphA2 on HEK cells results in functional mimicry with the formation of EphA2 dimers (mediated by the dimerization domain and not the clustering interface) by two different fluorescence methods and mutagenesis. They then demonstrate that binding of the glycoprotein cause cell shrinkage as expected from binding the authentic ligand. Thus, glycoprotein binding fully mimics ligand binding.

The paper is well written with an extensive introduction and discussion and a plethora of information provided in the supplementary material. The entire first section of the results and the role and demonstration of important residues for binding the glycoprotein to the EphA2 of residues was published by another group last year (Su C, Wu L, Chai Y, Qi J, Tan S, Gao GF, Song H, Yan J. Molecular basis of EphA2 recognition by gHgL from gammaherpesviruses. Nat Commun. 2020 Nov 24;11(1):5964. doi: 10.1038/s41467-020-19617-9. PMID: 33235207; PMCID: PMC7687889). The authors reference this work in the introduction, but do not refer to it in comparing their structure with this previously reported structure. Presumably, because they do not highlight differences, the two structures are in close agreement as suggested by the overlap of residues mutated to confirm their role in binding.

As far as I can tell the cellular work described is the novel and important contribution of the present paper. While I am not an expert in the fluorescence experiments described, the results appear to be internally consistent, and it is very likely that the glycoprotein mimicry to the ligand binding is very strong. It is unfortunate that the implications for virus infection of this mimicry was not further explored. That would be of great interest and enhance the novelty of the present work. Presumably that is work currently in progress.

We completely agree and we were eager to collect the data on how the EphA2 mutations impact HHV8 entry using the infection assay established in the Neipel lab. But due to the

pandemic and the research staff having to shift to work on covid related projects, we were not able to gather all the necessary data. The very preliminary results suggest that the same trend we observe in FSI-FRET experiments with soluble gH/gL and that is that the EphA2 with mutations in the CIN does not affect HHV8 infection much, while the EphA2 impaired in the DIN significantly decreases the infection. It is our priority and great interest to conclude these studies, but we decided not to wait for them because we wanted to ensure to submit the manuscript within the 6 months 'scoop grace period'.

The paper will be improved with attention to the following.

1. There should be some comparison of their structure with the previously reported structure so that their close similarity does not have to be implied. E.g. are they in the same space group, if not, do crystal contacts effect any of the regions that were disordered in the current structure etc. Regardless, are same regions disordered in both structures?

We agree with the comment. The reason we did not comment sufficiently on the structure reported by Su *et al.* is that the two are indeed very similar. But we have now added the text (lines 148-154) and a panel in S3B Fig. to illustrate the structural similarity so that it doesn't have to be implied. The new observations that were not made before were also emphasized to stand out clearly in the Discussion

2. A great deal of the discussion is a restatement of the results. The paper could be significantly shortened by a "results and discussion" section.

We agree, and we have shortened the Discussion by removing the text that was restating the results, and restructured it to contain subsections to highlight the principal findings. We think that combining Results and Discussion is a good idea, but because the 3 other reviewers asked for condensing the Discussion, rather than combining it with Results, we decided to keep the Results and Discussion separate.

3. There are a few minor points that should be clarified below.

Line 49 "As other herpesviruses, HHV-8 attaches to cells via its envelope glycoproteins…." Present in the tegument?

We are not completely sure we understand the comment, but to be more clear we modified this sentence to "*As other herpesviruses, HHV-8 attaches to cells via its glycoproteins that protrude from the virus surface and engage in numerous low affinity interactions with ubiquitous cellular factors such as heparan sulfate proteoglycans (6)*"

Line 66 "Despite low gH and gL sequence conservation across the herpesvirus family, the structures are remarkably similar, indicating conservation of a function." It would help to clarify if gH and gL are similar to each other or if gH is similar to other gH molecules and gL is similar to other gL molecules.

We agree - the text has been modified "*Despite low gH and gL sequence conservation across the herpesvirus family, the structures of gH/gL complexes from different herpesviruses are remarkably similar, indicating conservation of a function.*"

Line 146 "south" should be sought

The error has been fixed.

Line 233 "The introduced sites N30gL and N68 gL were glycosylated as clearly observed by gL shift to a higher molecular weight on SDS-PAGE gels…." It is not clear if the N residues were inserted of if they replaced another residue in which case this should be designated X30NgL etc. This should be clarified.

The N-glycosylation sites were introduced by one or two point substitutions designed to create the NXS/T N-glycosylation motif. We edited the text to reflect the type of changes introduced, and hope it is more clear now.
