# Supplementary material for: Human herpesvirus 8 molecular mimicry of ephrin ligands facilitates cell entry and triggers EphA2 signaling
Source: PLoS Biol. 2021 Sep 9;19(9):e3001392. doi: 10.1371/journal.pbio.3001392 (PMC8454987; doi:10.1371/journal.pbio.3001392)
Supplement: Attachment — Submitted filename: Response to the Editorial Requests.pdf [file pbio.3001392.s022.pdf]

Response to the Editorial Requests

Dear Dr Hodge,

Please find our replies (in blue) to the points you raised (in black) in text below.

Kind regards,
Marija Backovic

Dear Dr Backovic,

Thank you for submitting your revised Research Article entitled "Human herpesvirus 8 molecular mimicry of ephrin ligands facilitates cell entry and triggers EphA2 signaling" for publication in PLOS Biology.

I have now obtained advice from the Academic Editor handling your submission. Based on the your responses to the reviewer comments, we will probably accept this manuscript for publication, provided you satisfactorily address the following data and other policy-related requests that I have outlined below.

As you address these items, please take this last chance to review your reference list to ensure that it is complete and correct. If you have cited papers that have been retracted, please include the rationale for doing so in the manuscript text, or remove these references and replace them with relevant current references. Any changes to the reference list should be mentioned in the cover letter that accompanies your revised manuscript.

We have not changed any references.

A) You may be aware of the PLOS Data Policy, which requires that all data be made available without restriction:https://urldefense.com/v3/__http://journals.plos.org/plosbiology/s/data-availability__;!!JFdNOqOXpB6UZW0!6gJkRlK6f1QBGLoHyDzjHNy-PGaXyvFNjmtLLUzXXVtBgfZ4uA7TrefCBUJcA-NMYpYpca-o$. For more information, please also see this editorial:https://urldefense.com/v3/__http://dx.doi.org/10.1371/journal.pbio.1001797__;!!JFdNOqOXpB6UZW0!6gJkRlK6f1QBGLoHyDzjHNy-PGaXyvFNjmtLLUzXXVtBgfZ4uA7TrefCBUJcA-NMYnMroTVH$

Note that we do not require all raw data. Rather, we ask that all individual quantitative observations that underlie the data summarized in the figures and results of your paper be made available in one of the following forms:

1) Supplementary files (e.g., excel). Please ensure that all data files are uploaded as 'Supporting Information' and are invariably referred to (in the manuscript, figure legends, and the Description field when uploading your files) using the following format verbatim: S1 Data, S2 Data, etc. Multiple panels of a single or even several figures can be included as multiple sheets in one excel file that is saved using exactly the following convention: S1_Data.xlsx (using an underscore).

2) Deposition in a publicly available repository. Please also provide the accession code or a reviewer link so that we may view your data before publication.

Regardless of the method selected, please ensure that you provide the individual numerical values that underlie the summary data displayed in the following Figures, as they are essential for readers to assess your analysis and to reproduce it.

Fig 2C-D, 4A-D, 5A-D, 6B, 6D, Fig S4, S6, S9A-B, S10A-N.

NOTE: the numerical data provided should include all replicates AND the way in which the plotted mean and errors were derived (it should not present only the mean/average values).

We followed method 1) and have compiled all the numerical data in two files that are uploaded:

S1_data.xls contains the data related to the figures in the main text (Fig 2C-D, 4A-D, 5A-D, 6B and 6D)
S2_data.xls contains the data related to the figures in the Supporting Information (Fig S4, S6, S9A-B, S10)

Each excel file contains sheets labeled by the Figure / panel number the data correspond to.

B) Please also ensure that each of the relevant figure legends in your manuscript include information on *WHERE THE UNDERLYING DATA CAN BE FOUND*, and ensure your supplemental data file/s has a legend.

We have included reference to where the underlaying data are found in the legends of Figs 2C-D, 4A-D, 5A-D, 6B, 6D, Fig S4, S6, S9A-B, S10A-N. You will see that in the manuscript *doc file where the changes tracked on.

C) We require the original, uncropped and minimally adjusted image supporting the Western blot result reported in Figure S7 in the Supplementary Information file. We will require these files before a manuscript can be accepted so please prepare and upload them now. Please carefully read our guidelines for how to prepare and upload this data: https://urldefense.com/v3/__https://journals.plos.org/plosbiology/s/figures*loc-blot-and-gel-reporting-requirements__;Iw!!JFdNOqOXpB6UZW0!6gJkRlK6f1QBGLoHyDzjHNy-PGaXyvFNjmtLLUzXXVtBgfZ4uA7TrefCBUJcA-NMYjqlDvh7$

We have submitted the original image of the Western blot as a file under "Supplementary Information", labeled as S7_raw_image.pdf

We expect to receive your revised manuscript within two weeks.

To submit your revision, please go to https://urldefense.com/v3/__https://www.editorialmanager.com/pbiology/__;!!JFdNOqOXpB6UZW0!6gJkRlK6f1QBGLoHyDzjHNy-PGaXyvFNjmtLLUzXXVtBgfZ4uA7TrefCBUJcA-NMYpZM-P4g$ and log in as an Author. Click the link labelled 'Submissions Needing Revision' to find your submission record. Your revised submission must include the following:

- a cover letter that should detail your responses to any editorial requests, if applicable, and whether changes have been made to the reference list
- a Response to Reviewers file that provides a detailed response to the reviewers' comments (if applicable)
- a track-changes file indicating any changes that you have made to the manuscript.

In addition to this cover letter we are providing a track-changes *doc file of the revised manuscript.

NOTE: If Supporting Information files are included with your article, note that these are not copyedited and will be published as they are submitted. Please ensure that these files are legible and of high quality (at least 300 dpi) in an easily accessible file format. For this reason, please be aware that any references listed in an SI file will not be indexed. For more information, see our Supporting Information guidelines: https://urldefense.com/v3/__https://journals.plos.org/plosbiology/s/supporting-

information__;!!JFdNOqOXpB6UZW0!6gJkRlK6f1QBGLoHyDzjHNy-PGaXyvFNjmtLLUzXXVtBgfZ4uA7TrefCBUJcA-NMYve8B5Vx$

We have prepared Supporting Information: S1-S12 Figures, S1-S3 Tables, S1-S2 Text and S1-S2 Data, and uploaded them as separate files, with the figure legends at the end of the manuscript, after the Reference section.

Published Peer Review History
Please note that you may have the opportunity to make the peer review history publicly available. The record will include editor decision letters (with reviews) and your responses to reviewer comments. If eligible, we will contact you to opt in or out. Please see here for more details:
https://urldefense.com/v3/__https://blogs.plos.org/plos/2019/05/plos-journals-now-open-for-published-peer-review/__;!!JFdNOqOXpB6UZW0!6gJkRlK6f1QBGLoHyDzjHNy-PGaXyvFNjmtLLUzXXVtBgfZ4uA7TrefCBUJcA-NMYl6M2NT_$

We would be interested to have the Peer Review History published.

Early Version
Please note that an uncorrected proof of your manuscript will be published online ahead of the final version, unless you opted out when submitting your manuscript. If, for any reason, you do not want an earlier version of your manuscript published online, uncheck the box. Should you, your institution's press office or the journal office choose to press release your paper, you will automatically be opted out of early publication. We ask that you notify us as soon as possible if you or your institution is planning to press release the article.

We do want an early version of the manuscript.

Protocols deposition
To enhance the reproducibility of your results, we recommend that if applicable you deposit your laboratory protocols in protocols.io, where a protocol can be assigned its own identifier (DOI) such that it can be cited independently in the future. Additionally, PLOS ONE offers an option for publishing peer-reviewed Lab Protocol articles, which describe protocols hosted on protocols.io. Read more information on sharing protocols athttps://urldefense.com/v3/__https://plos.org/protocols?utm_medium=editorial-email&utm_source=authorletters&utm_campaign=protocols__;!!JFdNOqOXpB6UZW0!6gJkRlK6f1QBGLoHyDzjHNy-PGaXyvFNjmtLLUzXXVtBgfZ4uA7TrefCBUJcA-NMYhqZRkwH$

We will consider this.

Thank you!

Marija

Please do not hesitate to contact me should you have any questions.

Sincerely,

Richard

Richard Hodge, PhD
Associate Editor, PLOS Biology
rhodge@plos.org

PLOS
Empowering researchers to transform science

Carlyle House, Carlyle Road, Cambridge, CB4 3DN, United Kingdom
ORCiD I plosbio.org I @PLOSBiology I Blog

California (U.S.) corporation #C2354500, based in San Francisco
